# Studies toward synthesis of the core skeleton of spiroaspertrione A

**DOI:** 10.3389/fchem.2022.1022533

**Published:** 2022-10-05

**Authors:** Zhong-Hui Shen, Si-Yuan Lu, Jing-Yun Zheng, Xiang-Zhi Zhang, Jin-Bao Peng, Ai-Jun Ma

**Affiliations:** School of Biotechnology and Health Sciences, Wuyi University, Jiangmen, China

**Keywords:** studies, synthesis, core skeleton, spiroaspertrione A, natural product

## Abstract

Bioassay-guided isolation of spiroaspertrione A from cultures of *Aspergillus sp*. TJ23 in 2017 demonstrated potent resensitization of oxacillin against methicillin-resistant *Staphylococcus aureus* by lowering the oxacillin minimal inhibitory concentration up to 32-fold. To construct this unique spiro[bicyclo[3.2.2]nonane-2,1′-cyclohexane] system, a protocol for ceric ammonium nitrate-induced intramolecular cross-coupling of silyl enolate is disclosed.

## Introduction

The expansion of multidrug-resistant pathogens is a threat to human health that can effectively take us back to the pre-antibiotic era for many infectious diseases (Walsh et al., 2011). Considering its grave roles in hospital and community-acquired infections, methicillin-resistant *Staphylococcus aureus* (MRSA) is a “superbug” with an extreme array of resistance and virulence factors (Gonzales, et al., 2015). Drug-resistance gene mutations of MRSA are exemplified by *mecA*, the disruption of which can produce inducible resistance to *β*-lactam antibiotics because it encodes penicillin-binding protein 2a (Fuda et al., 2005). With the rapid acquisition of resistance restricting therapeutic options for MRSA, many scientists have explored treatment methods combining the use of small molecules to render MRSA sensitive to the effects of conventional *β*-lactam antibiotics (Van Hal et al., 2011; Long et al., 2014; Bush, 2015; Gonzales et al., 2015).

**FIGURE 1:**
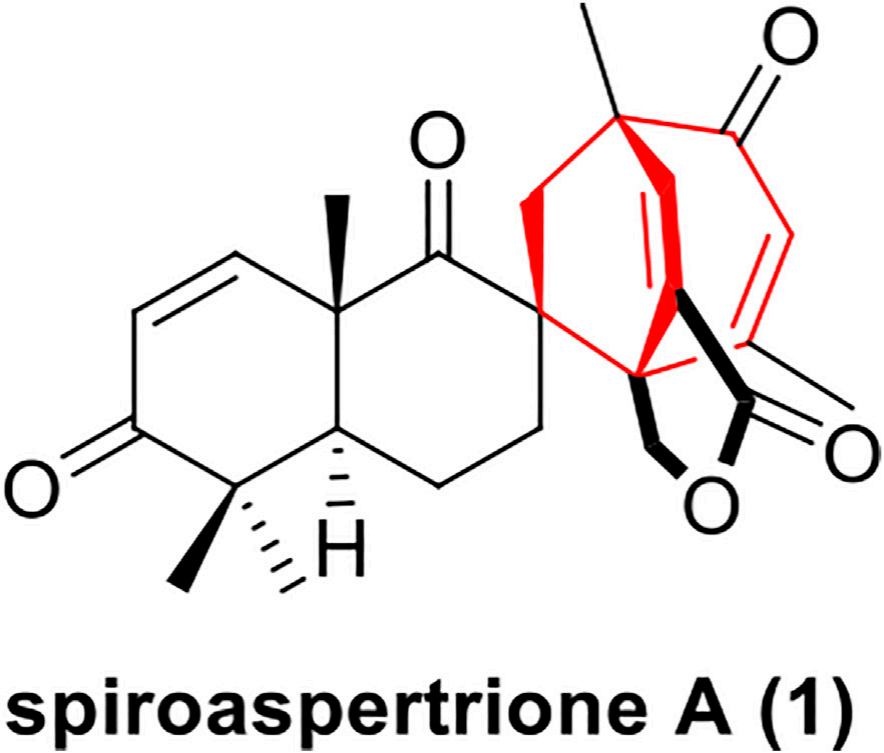
Structure of spiroaspertrione A (1).

In 2017, Zhang group used a bioassay-guided approach to isolate a novel terpene-polyketide hybrid spiromero-terpenoid from a culture of *Aspergillus sp.* TJ23, spiroaspertrione A **(1)**, which bears a unique spiro[bicyclo[3.2.2]nonane-2,1′- cyclohexane] carbocyclic skeleton (Figure 1) (He et al., 2017). Spiroaspertrione A demonstrated potent resensitization of oxacillin against MRSA by lowering the oxacillin minimal inhibitory concentration (MIC) up to 32-fold from 32 μg/mL to 1 μg/mL (He et al., 2017). This promising bioactivity together with a unique spiro[bicyclo[3.2.2]nonane-2,1-cyclohexane] carbocyclic skeleton renders spiroaspertrione A an interesting and challenging target for total synthesis. To date, no synthesis method for spiroaspertrione A has been reported.

**FIGURE 2:**
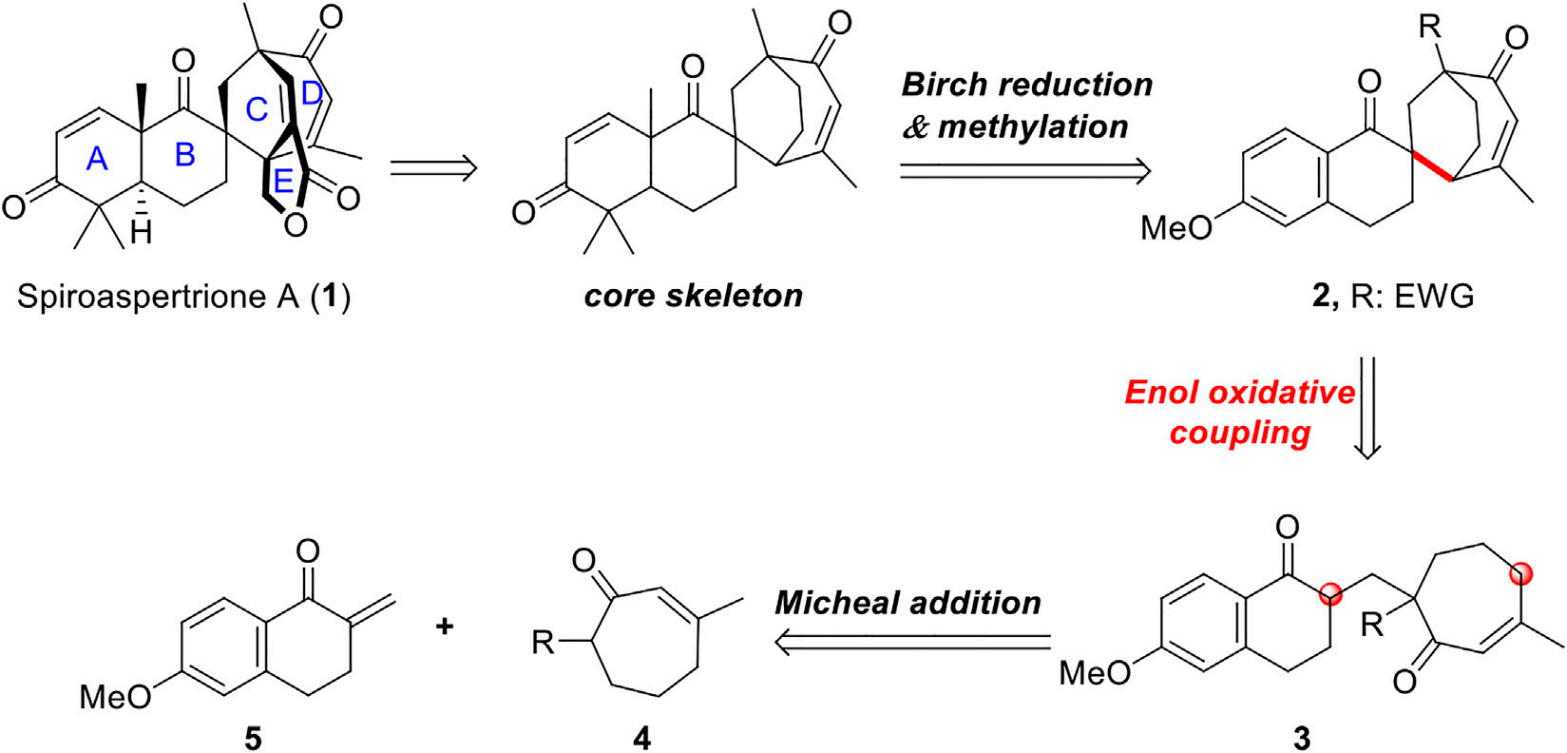
Retrosynthetic analysis.

Here, we analyzed and constructed the core skeleton of this spiromeroterpenoid, culminating in a strategy of intramolecular enol oxidative coupling (Figure 2). When designing this synthetic strategy for spiroaspertrione A, we noticed that the E lactone ring can be obtained by simple lactonization during later synthesis. Construction of the spiro[bicyclo[3.2.2]nonane] system of the ABCD rings exhibits a high degree of ring tension and rigidity–the most interesting and challenging feature of spiroaspertrione A synthesis. The core skeleton of spiroaspertrione A could be constructed through *Birch* reduction followed by methylation of the naphthene compound 2. This synthetically significant and more tractable spiro-ring system can then be built by an intramolecular enol oxidative coupling (EOC) reaction of precursor 3, which can then be traced back to a 1,4-conjugate addition of western fragment 5 and eastern fragment 4.

**FIGURE 3:**
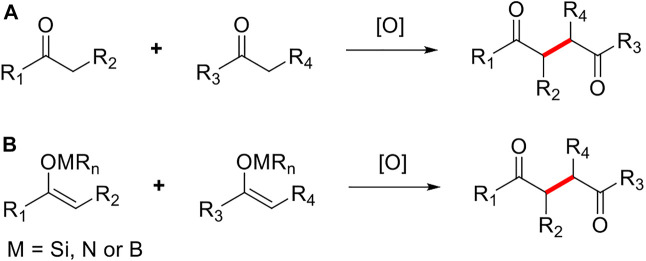
Types of enol oxidative coupling reaction.

As an efficient synthetic method to directly construct C-C bonds, the oxidative coupling reaction of enol derivatives has been applied in the syntheses of polyketides, alkaloids, and other natural products. (Murarka and Antonchick 2018). Although the first oxidative coupling reaction of enol derivatives dates back to 1935, it did not receive widespread attention from chemists until the 1970s because the efficiency and practicality of this reaction were less than satisfactory (Fujii et al., 1992; Kohno and Narasaka 1995; Ryter and Livinghouse 1998; Ekebergh et al., 2011; Rathke and Lindert 1971; Dessau and Heiba 1974; Xie and Huang 2010; Renaud and Fox 1998). In 2005, Baran group began to conduct in-depth research on the oxidative coupling reaction of enolates and successfully applied their findings to the total synthesis of multiple complex natural products (Baran et al., 2005; Richter et al., 2007; DeMartino et al., 2008). To date, the oxidative coupling reaction of enol derivates remains under constant development and optimization. The EOC reaction can be broadly divided into two categories: direct oxidation, involving the construction of C-C bonds under single-electron oxidants (e.g., ketones, carboxylic acids, esters, and amides) bound to the corresponding enols or enolates; and indirect oxidation, in which single-electron oxidants are converted to the corresponding enol (e.g., silanes and enamines) prior to construction of the C-C bonds (Figure 3). The EOC reaction has been reviewed by Plumet (Csákÿ and Plumet 2001), Baran (Baran 2006), Dong (Yeung Dong 2011), Thomson (Guo et al., 2012), Ma (Nagaraju and Ma 2018), Chen (Chen and Liu 2021), and others.

**FIGURE 4:**
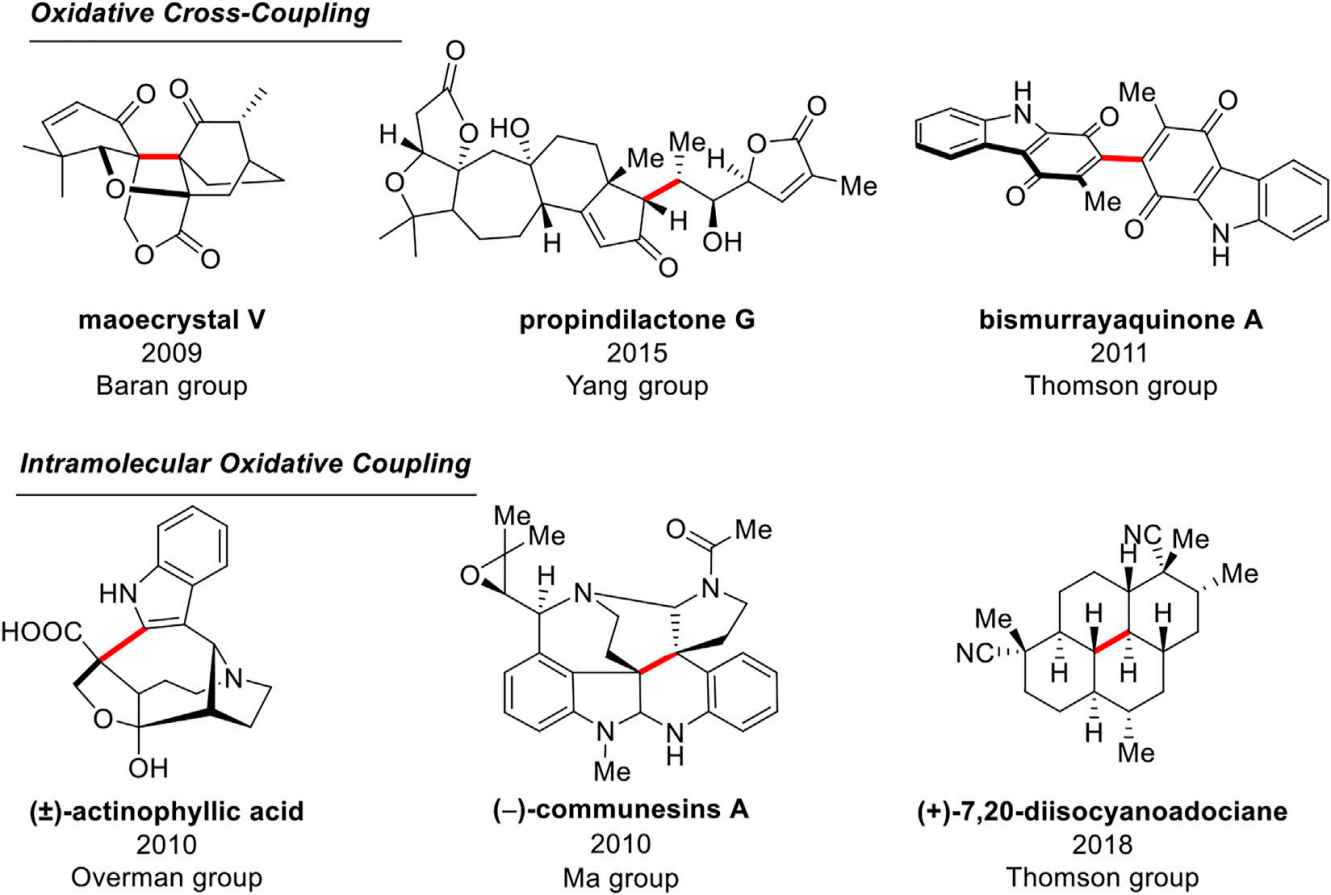
Application of EOC reaction in the synthesis of natural products.

In recent years, intermolecular and intramolecular EOC reactions have been applied to many natural products as an efficient method of constructing C-C bonds (Figure 4). Baran group completed the construction of the core skeleton of the natural product *maoecrystal* V using intermolecular EOC reactions (Krawczuk et al., 2009). Furthermore, Yang group (You et al., 2015) used enol silyl ethers substrates to realize the enantioselective synthesis of *propindilactone* G from the Schisandra family by cross-oxidative coupling reaction. Moreover, Thomson groups disclosed a method of *self-*intermolecular EOC reactions applied to the synthesis of dimerized natural product *bis-murrayaquinone* A (Konkol et al., 2011).

Using intramolecular EOC reactions, Overman group (Martin et al., 2008; Martin et al., 2010) made important progress toward the total synthesis of the indole alkaloids (±)-*actinophyllic acid*. From 2010 to 2014, Ma group (Zuo et al., 2010; Zuo and Ma 2011; Zi et al., 2012; Wei et al., 2013; Teng et al., 2014) realized the efficient construction of the core skeleton of indole alkaloids and synthesized several indole alkaloids such as (+)-*communesins* A. In 2018, Thomson group (Guo et al., 2012; Jones et al., 2014; Robinson and Thomson, 2018) reported the strategy of *intra*-EOC reaction using enol *di*-silyl ether to realize the formal synthesis of natural products (**+**)**-**7,20-*diisocyanoadociane* and other derived products.

## Materials and methods

Unless otherwise noted, all reactions were carried out under N_2_ atmosphere. All reagents were from commercial sources and used as received without further purification. All solvents were dried by standard techniques and distilled prior to use. Column chromatography was performed on silica gel (200–300 meshes) using petrol etherand ethyl acetate as eluent. NMR spectra were recorded on a Bruker Avance operating at for ^1^H NMR at 500 MHz, ^13^C NMR at 126 MHz and spectral data were reported in ppm relative to tetramethylsilane (TMS) as internal standard and CDCl_3_ (^1^H NMR δ 7.26, ^13^C NMR δ 77.0) as solvent. All high-resolution mass spectra (HRMS) were obtained by Thermo Scientific's UltiMate 3,000 Series liquid system and Thermo Scientific Q-Exactive combined quadrupole Orbitrap mass spectrometer.

**FIGURE 5:**
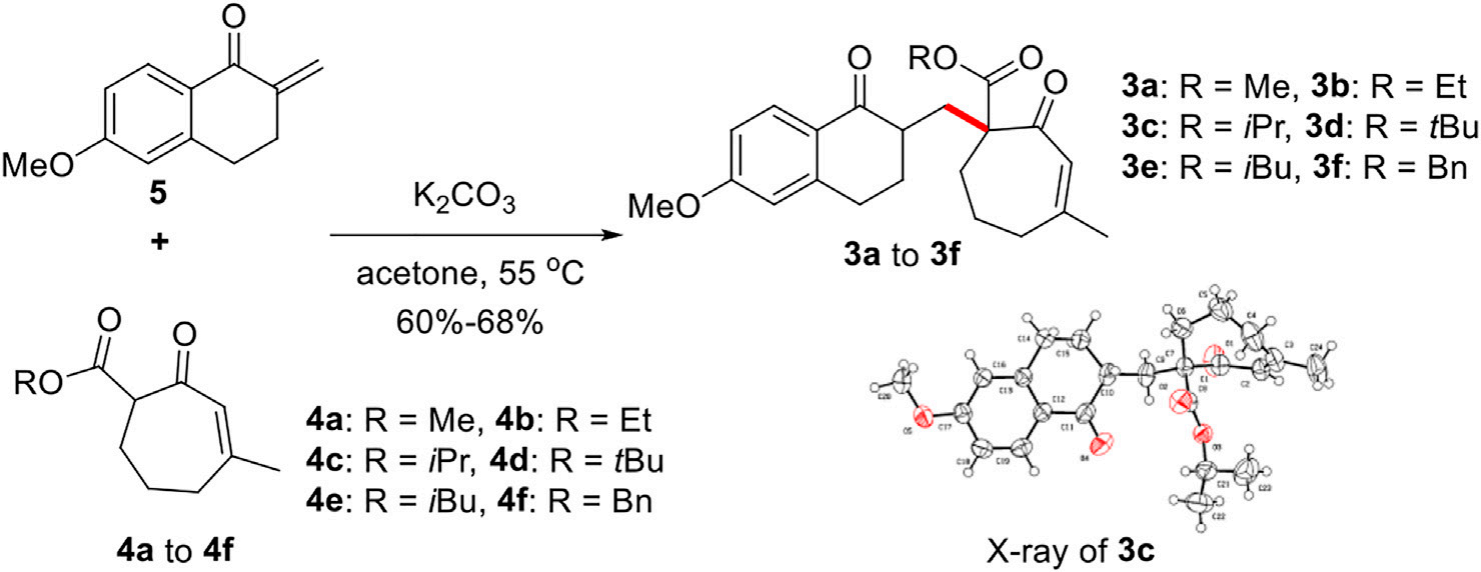
Synthesis of precursor 3.

According to our retrosynthetic analysis, we chose known compound 5 (Li et al., 2019) and 4 self-prepared from 3-methylcyclohept-2-en-1-one as substrates to form the important precursor 3. Following screening of various conditions, we obtained compound 3 as a minor product with a yield of 30% under NaH and MeOH, and compound 5’s *O*-1,4-addition byproduct as the major product. Subsequent screening of several Lewis acids, such as BF_3_·OEt and TiCl_4_, yielded substrate 5’s *O*-DA reaction byproduct as the major product. To our delight, conducting the reaction in acetone at 55°C in the presence of K_2_CO_3_(2 eq) afforded the desired 1,4-addition product 3 with 60%–68% yield (Figure 5).

With precursor 3a in hand, we intended to construct the desired C-C bond by single-electron oxidation under conditions including a metal base (Table 1). In the presence of LHMDS and cupric chloride (CuCl_2_) or ferric chloride (FeCl_3_), we obtained a very small amount of the EOC product 2a and 2a′, although the recovery yield was 60% (Table 1, entries 1 and 2). We assumed that LHMDS conditions were not conducive to the formation of stable enolates, thus, screened various metal bases. When using LDA as the base, the reaction only provided a complex mixture and trace amount of product with the oxidant CuCl_2_ (Table 1, entry 3). The substrate was completely consumed under conditions including KHMDS or NaHMDS (Table 1, entries 4 and 5). Although we tried numerous oxidative conditions, the yield of oxidative coupling products was not significantly improved (Table 1, entries 6 to 11).

**TABLE 1:** Optimization of the reaction conditions^a^.

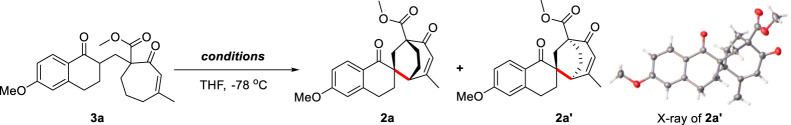		
Entry	Conditions	Yield (2a+2a′)
1	LHMDS, CuCl_2_	<5%, 60% (brsm)^b^
2	LHMDS, FeCl_3_	<5%, 53% (brsm)^b^
3	LDA, CuCl_2_	complex mixture
4	KHMDS, CuCl_2_	9%
5	NaHMDS, CuCl_2_	11%
6^c^	NaHMDS, CuCl_2_, O_2_	--
7^d^	NaHMDS, CuCl_2_, air	--
8	NaHMDS, Cu(acac)_2_	8%
9	NaHMDS, FeCl_3_	9%
10	NaHMDS, I_2_	--
11^e^	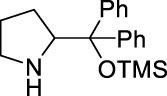 Cu(acac)_2_	--

a Reactions were carried out with 3a (30 mg, 0.081 mmol), metal base (0.243 mmol), and single-electricity oxidant (0.162 mmol) in THF (2.0 ml) under N_2_.

b Brsm = based on the recovered starting material.

c Reactions were carried out with CuCl_2_ (20% mmol) under O_2_.

d Reactions were carried out with CuCl_2_ (20% mmol) under air.

e Reactions were carried out with 10 mol% catalyst and Cu(acac)_2_ (20 mol%).

## Results and discussion

According to the unsatisfactory experimental results described above, we assumed that substrate 3a may form more stable metal complexes with Cu(II) or Fe(III) ions under the alkaline system, thereby inhibiting the process of oxidative coupling. Therefore, we envisaged the replacement of this stable complex by enol silyl ether (Table 2). We chose compound 3a as a substrate to first optimize the silyl bis-enol etherification condition. The desired silyl bis-enol ether product 6a was obtained with 21% yield in THF (2.0 ml) at -78°C under N_2_ in the presence of LHMDS (0.243 mmol) and TBSOTf (0.162 mmol) (Table 2, entry 2). As previously mentioned, it was not conducive to obtain silyl enol ethers and could be broken down using LDA as the base (Table 2, entry 1). Encouraged by this result, we surveyed other bases including NaHMDS, KHMDS and Et_3_N, and found that NaHMDS generated the best yield (58%) while Et_3_N only generated monosilyl product (Table 2, entries 3–6).

**TABLE 2:** Optimization of silyl bis-enol etherification conditions^a^.

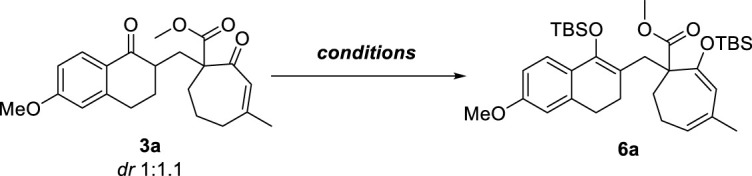			
Entry	Conditions	Yield %	
		Bis-silyl	Mono-silyl
1	LDA, TBSOTf, THF	complex mixture	
2	LHMDS, TBSOTf, THF	21	75
3^b^	LHMDS, HMPA, TBSCl, THF	24	74
4	KHMDS, TBSOTf, THF	35	60
5	NaHMDS, TBSOTf, THF	58	30
6^c^	Et_3_N, TBSOTf, DCM	0	90

a Reactions were carried out with 3a (30 mg, 0.081 mmol), base (0.243 mmol), and TBSOTf (0.162 mmol) in THF (2.0 ml) at -78°C under N_2._

b Reactions were carried out with 3a (30 mg, 0.081 mmol), LHMDS (0.243 mmol), TBSCl (0.162 mmol) and HMPA (0.162 mmol) in THF (2.0 ml) at -78°C under N_2._

c Reactions were carried out with 3a (30 mg, 0.081 mmol), Et_3_N (0.243 mmol), TBSOTf (0.162 mmol) in DCM (2.0 ml) at room temperature under N_2._

With the enol bis-silyl ether 6a in hand, we intended to optimize the intramolecular enol oxidative coupling reaction (Table 3). Conducting the reaction in CH_3_CN/THF at 0°C in the presence of CAN (0.24 mmol) and NaHCO_3_ (0.48 mmol) exclusively afforded the coupling products in 77% isolated yield after 0.5 h (Table 3, entry 1). However, the main product 4a′ identified by X-ray analyses was an undesired stereoisomer. We intended to optimize the diastereomeric ratio (*dr*) by changing the ester group of 6 (Table 3, entries 2–5) and found that the isopropyl *dr* of ester substrate (3c) reached 4.9:1. We also explored the effect of different silicon groups (Table 3, entries 6–7). Unfortunately, changing the silicon groups did not decisively progress the EOC reaction. Finally, we evaluated different oxidants to optimize the *dr* of products (Table 3, entries 8–12). Cu(II) chloride and Fe(III) chloride produced the coupling products with 21% and 23% yields, but even more of the desilylation product 3 (Table 3, entries 8 and 10). Other metal oxidants, including Cu(acac)_2_ and AgF, provided a complex mixture of product and raw product 6c (Table 3, entries 9 and 11), and the result of Koser’s reagent (PhI(OH)OTs) was also unsatisfactory (Table 3, entry 12).

**TABLE 3:** Optimization of intramolecular EOC reaction conditions^a^.

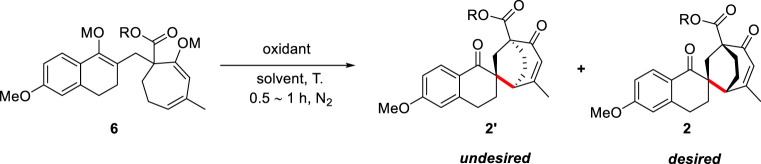							
Entry	R	M	Oxidant	Solvent	T (^o^C)	dr^b^	Yield^c^
1	Me	TBS	CAN	CH_3_CN/THF	0	8.7:1	77%
2	Et	TBS	CAN	CH_3_CN/THF	0	8:1	74%
**3**	***i*Pr**	**TBS**	**CAN**	**CH_3_CN/THF**	**0**	**4.9:1**	**69%**
4	*t*Bu	TBS	CAN	CH_3_CN/THF	0	12:1	68%
5	*i*Bu	TBS	CAN	CH_3_CN/THF	0	6.4:1	62%
6	*i*Pr	TES	CAN	CH_3_CN/THF	0	5.8:1	64%
7	*i*Pr	TIPS	CAN	CH_3_CN/THF	0	6.8:1	64%
8	*i*Pr	TBS	CuCl_2_	CH_3_CN/THF	–78 to rt	--	21% (50%)^d^
9	*i*Pr	TBS	Cu(acac)_2_	CH_3_CN/THF	–78 to rt	--	--
10	*i*Pr	TBS	FeCl_3_	CH_3_CN/THF	–78 to rt	--	23% (54%)^d^
11	*i*Pr	TBS	AgF, PhBr	CH_3_CN	Rt	--	no reaction
12	*i*Pr	TBS	PhI(OH)OTs	DCM	–78	12:1	61%

a Reactions were carried out with 6 (0.08 mmol), CAN (0.24 mmol), and NaHCO_3_ (0.48 mmol) in CH_3_CN/THF (0.1 M, 4:1) at 0°C under N_2._

b Determined ratio of 2′ and 2 by ^1^H NMR.

c Isolated yields of 2 and 2′ after purification by column chromatography.

d Isolated yields of 3 after purification by column chromatography.

## Conclusion

In conclusion, we developed an efficient method of constructing the spiro[bicyclo[3.2.2]nonane] system by intramolecular enol oxidative coupling reaction. Although the diastereomeric ratio of products is embarrassing, the high yield of this remote oxidative coupling reaction to build rigid spiro[bicyclo[3.2.2]nonane] structures is encouraging. Our findings once again confirm the practicality of enol oxidative coupling reactions in natural products and provide a new strategy for the synthesis of spiroaspertrione A. Further study for the total synthesis of spiroaspertrione A is underway in our laboratory.

## Data Availability

The datasets presented in this study can be found in online repositories. The names of the repository/repositories and accession number(s) can be found in the article/Supplementary Material.

## References

[B1] Baran P. S. Richter J. M. Lin D. W. (2005). Direct coupling of pyrroles with carbonyl compounds: Short enantioselective synthesis of (S)‐Ketorolac. Angew. Chem. 117, 615–618. 10.1002/ange.200462048 15584071

[B2] Baron P. S. (2006). Enantioselective total synthesis of avrainvillamide and the stephacidins. J. Am. Chem. Soc. 128, 8678–8693. 10.1021/ja061660s 16802835

[B3] Bush K. (2015). Synergistic MRSA combinations. Nat. Chem. Biol. 11, 832–833. 10.1038/nchembio.1935 26485079

[B4] Chen W. Liu Q. (2021). Recent advances in the oxidative coupling reaction of enol derivatives. Chin. J. Org. Chem. 41, 3414–3430. 10.6023/cjoc202104058

[B5] Csákÿ A. G. Plumet J. (2001). Stereoselective coupling of ketone and carboxylate enolates. Chem. Soc. Rev. 30, 313–320. 10.1039/B104000F

[B6] DeMartino M. P. Chen K. Baran P. S. (2008). Intermolecular enolate heterocoupling: Scope, mechanism, and application. J. Am. Chem. Soc. 130, 11546–11560. 10.1021/ja804159y 18680297

[B7] Dessau R. M. Heiba E. A. I. (1974). Oxidation by metal salts. XII. Novel one-step synthesis of 1, 4-diketones. J. Org. Chem. 93, 3457–3459. 10.1021/jo00937a053

[B8] Ekebergh A. Karlsson I. Mete R. Pan Y. Börje A. Mårtensson J. (2011). Oxidative coupling as a biomimetic approach to the synthesis of scytonemin. Org. Lett. 13, 4458–4461. 10.1021/ol201812n 21786790 PMC3164230

[B9] Fuda C. Hesek D. Lee M. Morio K. I. Nowak T. Mobashery S. (2005). Activation for catalysis of penicillin-binding protein 2a from methicillin-resistant Staphylococcus a ureus by bacterial cell wall. J. Am. Chem. Soc. 127, 2056–2057. 10.1021/ja0434376 15713078

[B10] Fujii T. Hirao T. Ohshiro Y. (1992). Oxovanadium-induced oxidative desilylation for the selective synthesis of 1, 4-diketones. Tetrahedron Lett. 33, 5823–5826. 10.1016/0040-4039(92)89041-A

[B11] Gonzales P. R. Pesesky M. W. Bouley R. Ballard A. Biddy B. A. Suckow M. A. (2015). Synergistic, collaterally sensitive β-lactam combinations suppress resistance in MRSA. Nat. Chem. Biol. 11, 855–861. 10.1038/nchembio.1911 26368589 PMC4618095

[B12] Guo F. Clift M. D. Thomson R. J. (2012). Oxidative coupling of enolates, enol silanes, and enamines: Methods and natural product synthesis. Eur. J. Org. Chem. 2012, 4881–4896. 10.1002/ejoc.201200665 PMC3586739 23471479

[B13] He Y. Hu Z. Sun W. Li Q. Li X. N. Zhu H. (2017). Spiroaspertrione A, a bridged spirocyclic meroterpenoid, as a potent potentiator of oxacillin against methicillin-resistant *Staphylococcus aureus* from *Aspergillus* sp. TJ23. J. Org. Chem. 82, 3125–3131. 10.1021/acs.joc.7b00056 28219242

[B14] Jones B. T. Avetta C. T. Thomson R. J. (2014). Total synthesis of propolisbenzofuran B. Chem. Sci. 5, 1794–1798. 10.1039/C4SC00356J 24976944 PMC4066987

[B16] Kise N. Tokioka K. Aoyama Y. Matsumura Y. (1995). Enantioselective synthesis of 2, 3-disubstituted succinic acids by oxidative homocoupling of optically active 3-acyl-2-oxazolidones. J. Org. Chem. 60, 1100–1101. 10.1021/jo00110a003

[B17] Kohno Y. Narasaka K. (1995). Oxidative generation of α-radicals of carbonyl compounds from the α-stannyl derivatives and their reactions with electron-rich olefins. Bull. Chem. Soc. Jpn. 68, 322–329. 10.1246/bcsj.68.322

[B18] Konkol L. C. Guo F. Sarjeant A. A. Thomson R. J. (2011). Enantioselective total synthesis and studies into the configurational stability of bismurrayaquinone A. Angew. Chem. Int. Ed. 50, 9931–9934. 10.1002/anie.201104726 PMC3517045 21898740

[B19] Krawczuk P. J. Schöne N. Baran P. S. (2009). A synthesis of the carbon skeleton of maoecrystal V. Org. Lett. 11, 4774–4776. 10.1021/ol901963v 19795876 PMC2783297

[B20] Li Y. P. Li Z. Q. Zhou B. Li M. L. Xue X. S. Zhu S. F. (2019). Chiral spiro phosphoric acid-catalyzed friedel–crafts conjugate addition/enantioselective protonation reactions. ACS Catal. 9, 6522–6529. 10.1021/acscatal.9b01502

[B21] Long S. W. Olsen R. J. Mehta S. C. Palzkill T. Cernoch P. L. Perez K. K. (2014). PBP2a mutations causing high-level ceftaroline resistance in clinical methicillin-resistant *Staphylococcus aureus* isolates. Antimicrob. Agents Chemother. 58, 6668–6674. 10.1128/AAC.03622-14 25155594 PMC4249384

[B22] Martin C. L. Overman L. E. Rohde J. M. (2008). Total synthesis of (±)-actinophyllic acid. J. Am. Chem. Soc. 130, 7568–7569. 10.1021/ja803158y 18491907 PMC2654095

[B23] Martin C. L. Overman L. E. Rohde J. M. (2010). Total synthesis of (±)-and (−)-actinophyllic acid. J. Am. Chem. Soc. 132, 4894–4906. 10.1021/ja100178u 20218696 PMC2851836

[B24] Murarka S. Antonchick A. P. (2018). Metal-catalyzed oxidative coupling of ketones and ketone enolates. Synthesis 50, 2150–2162. 10.1055/s-0037-1609715

[B25] Nagaraju K. Ma D. (2018). Oxidative coupling strategies for the synthesis of indole alkaloids. Chem. Soc. Rev. 47, 8018–8029. 10.1039/C8CS00305J 30221274

[B27] Rathke M. W. Lindert A. (1971). Reaction of ester enolates with copper (II) salts. Synthesis of substituted succinate esters. J. Am. Chem. Soc. 93, 4605–4606. 10.1021/ja00747a051

[B28] Renaud P. Fox M. A. (1988). Reaction of dilithiated carboxylic acids with iodine: Evidence for the formation of a radical anion intermediate. J. Org. Chem. 53, 3745–3752. 10.1021/jo00251a015

[B29] Richter J. M. Whitefield B. W. Maimone T. J. Lin D. W. Castroviejo M. P. Baran P. S. (2007). Scope and mechanism of direct indole and pyrrole couplings adjacent to carbonyl compounds: Total synthesis of acremoauxin A and oxazinin 3. J. Am. Chem. Soc. 129, 12857–12869. 10.1021/ja074392m 17900115 PMC2631414

[B30] Robinson E. E. Thomson R. J. (2018). A strategy for the convergent and stereoselective assembly of polycyclic molecules. J. Am. Chem. Soc. 140, 1956–1965. 10.1021/jacs.7b13234 29309727

[B31] Ryter K. Livinghouse T. (1998). Dichloro (2, 2, 2-trifluoroethoxy) oxovanadium (V). A remarkably effective reagent for promoting one-electron oxidative cyclization and unsymmetrical coupling of silyl enol ethers. J. Am. Chem. Soc. 120, 2658–2659. 10.1021/ja973585e

[B32] Teng M. Zi W. Ma D. (2014). Total synthesis of the monoterpenoid indole alkaloid (±)-Aspidophylline A. Angew. Chem. Int. Ed. 53, 1814–1817. 10.1002/anie.201310928 24481917

[B33] Van Hal S. J. Paterson D. L. Gosbell I. B. (2011). Emergence of daptomycin resistance following vancomycin-unresponsive *Staphylococcus aureus* bacteraemia in a daptomycin-naïve patient—A review of the literature. Eur. J. Clin. Microbiol. Infect. Dis. 30, 603–610. 10.1007/s10096-010-1128-3 21191627

[B34] Walsh T. R. Weeks J. Livermore D. M. Toleman M. A. (2011). Dissemination of NDM-1 positive bacteria in the New Delhi environment and its implications for human health: An environmental point prevalence study. Lancet Infect. Dis. 11, 355–362. 10.1016/S1473-3099(11)70059-7 21478057

[B35] Wei Y. Zhao D. Ma D. (2013). Total synthesis of the indole alkaloid (±)-and (+)-Methyl N-decarbomethoxychanofruticosinate. Angew. Chem. 125, 13226–13229. 10.1002/ange.201307788 24258849

[B36] Xie J. Huang Z. Z. (2010). The cascade carbo-carbonylation of unactivated alkenes catalyzed by an organocatalyst and a transition metal catalyst: A facile approach to γ-diketones and γ-carbonyl aldehydes from arylalkenes under air. Chem. Commun. 46, 1947–1949. 10.1039/B921310D 20198262

[B37] Yeung C. S. Dong V. M. (2011). Catalytic dehydrogenative cross-coupling: Forming carbon− carbon bonds by oxidizing two carbon-hydrogen bonds. Chem. Rev. 111, 1215–1292. 10.1021/cr100280d 21391561

[B38] You L. Liang X. T. Xu L. M. Wang Y. F. Zhang J. J. Su Q. (2015). Asymmetric total synthesis of propindilactone G. J. Am. Chem. Soc. 137, 10120–10123. 10.1021/jacs.5b06480 26181605

[B39] Zi W. Xie W. Ma D. (2012). Total synthesis of akuammiline alkaloid (−)-vincorine via intramolecular oxidative coupling. J. Am. Chem. Soc. 134, 9126–9129. 10.1021/ja303602f 22616754

[B40] Zuo Z. Ma D. (2011). Enantioselective total syntheses of communesins A and B. Angew. Chem. Int. Ed. 50, 12008–12011. 10.1002/anie.201106205 22006672

[B41] Zuo Z. Xie W. Ma D. (2010). Total synthesis and absolute stereochemical assignment of (−)-communesin F. J. Am. Chem. Soc. 132, 13226–13228. 10.1021/ja106739g 20812683

