# Supplementary material for: Studies toward synthesis of the core skeleton of spiroaspertrione A
Source: Front Chem. 2022 Oct 5;10:1022533. doi: 10.3389/fchem.2022.1022533 (PMC9581311; doi:10.3389/fchem.2022.1022533)
Supplement: Supplementary file 1 [file DataSheet1.PDF]

Supporting Information

Studies toward Synthesis of the Core Skeleton of Spiroaspertrione A

*Zhong-Hui Shen, Si-Yuan Lu, Jing-Yun Zheng, Xiang-Zhi Zhang, Jin-Bao Peng, Ai-Jun Ma**

School of Biotechnology and Health Sciences, Wuyi University, Jiangmen, Guangdong, 529020, People's Republic of China.

E-mail: wyuchemmaj@126.com

Table of Contents

General Information 1

Experimental Procedures 7

X-Ray Crystallographic Data 7

References.. 9

NMR Spectra 10

General Information

Unless otherwise noted, all reactions were carried out under N_2 atmosphere. All reagents were from commercial sources and used as received without further purification. All solvents were dried by standard techniques and distilled prior to use. Column chromatography was performed on silica gel (200-300 meshes) using petrol etherand ethyl acetate as eluent. NMR spectra were recorded on a Bruker Avance operating at for ^{1}H NMR at 500 MHz, ^{13}C NMR at 126 MHz and spectral data were reported in ppm relative to tetramethylsilane (TMS) as internal standard and CDCl3 (^{1}H NMR δ 7.26, ^{13}C NMR δ 77.0) as solvent. All high resolution mass spectra (HRMS) were obtained by Thermo Scientific's UltiMate 3000 Series liquid system and Thermo Scientific Q-Exactive combined quadrupole Orbitrap mass spectrometer. All coupling constants (J) are reported in Hz. The following abbreviations were used to describe peak splitting patterns when appropriate: s = singlet, d = doublet, dd = double doublet, ddd = double doublet of doublets, t = triplet, dt = double triplet, q = quatriplet, m = multiplet.

Experimental Procedures

1. General Procedure for Synthesis of Compound 4[1]

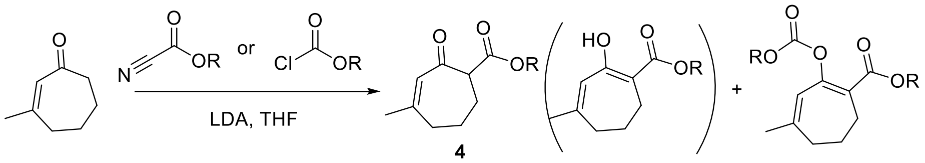

To a solution of 3-methylcyclohept-2-en-1-one (1.61 mmol) in THF (4 mL) was added LDA (2M in THF, 1.6 mL, 3.22 mmol) dropwise at -78°C. After stirring for 1 hour at -78 °C, mander’s reagent or chloroformate reagent (2.42 mmol) was added and the mixture was stirred for another 1 hour at -78 °C. Aq. sat $NaHCO_3$ (2 mL) and water was added, the aqueous layer extracted with EtOAc (3 × 5 mL). The organic combined layers were washed with brine (3 × 3 mL), dried over Na_2SO_4 and concentrated in vacuo. Further purification by Alkaline Alumina (PE : EtOAc, 80:1) to obtain mixture **4**.

2. General Procedure for Synthesis of Compound 3

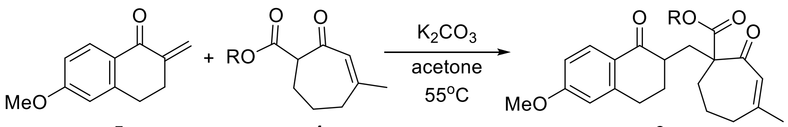

5 **4** **3**

To a solution of known compound **5**[2] (200 mg, 1.06 mmol, 1.0 equiv.) and mixture **4** (1.06 mmol, 1.0 equiv.) in acetone (5.3 mL) at room temperature, was added K_2CO_3 (293 mg, 2.12 mmol, 2.0 equiv.). The mixture was allowed to stir and warm to 55°C over night. The reaction mixture was diluted with aq. sat NH_4Cl (5 mL) and the aqueous layer extracted with EtOAc (3 × 5 mL). The combined layers were washed with brine (15 mL), dried over Na_2SO_4 and concentrated in vacuo. Flash chromatography (PE : EtOAc, 10:1) furnished coupling product **3** as a yellow oil.

Methyl 1-((6-methoxy-1-oxo-1,2,3,4-tetrahydronaphthalen-2-yl)methyl)-4-methyl-2-oxocyclohept-3-ene-1-carboxylate (**3a**)

^{1}H NMR (500 MHz, Chloroform-d) δ 7.9 (dd, *J* = 8.74, 5.69 Hz, 1H), 6.8 – 6.7 (m, 1H), 6.6 (t, *J* = 3.06 Hz, 1H), 5.9 (d, *J* = 38.36 Hz, 1H), 3.8 (d, *J* = 1.73 Hz, 3H), 3.7 (d, *J* = 12.13 Hz, 3H), 3.0 – 2.8 (m, 2H), 2.7 – 2.6 (m, 1H), 2.6 – 2.4 (m, 3H), 2.3 – 2.1 (m, 2H), 2.0 – 1.9 (m, 1H), 1.9 – 1.8 (m, 5H), 1.8 – 1.7 (m, 2H).

^{13}C NMR (126 MHz, Chloroform-*d*) δ 200.7, 200.4, 198.6, 198.5, 173.8, 173.6, 163.3, 163.3, 154.6, 154.5, 146.4, 146.3, 129.9, 129.9, 128.6, 127.8, 126.1, 126.1, 113.1, 113.0, 112.3, 112.3, 63.4, 63.3, 55.4, 55.4, 52.3, 52.3, 44.0, 43.9, 36.8, 36.6, 36.5, 36.0, 32.4, 32.0, 31.3, 30.8, 29.5, 29.4, 26.8, 26.4, 24.1, 23.1.

HRMS (ESI): $C_{22}H_{26}O_5Na$ $[M+Na]^+$ calculated: 393.1678, found 393.1675.

Ethyl 1-((6-methoxy-1-oxo-1,2,3,4-tetrahydronaphthalen-2-yl)methyl)-4-methyl-2-oxocyclohept-3-ene-1-carboxylate(3b)

^{1}H NMR (500 MHz, Chloroform-*d*) δ 7.9 (dd, *J* = 8.72, 5.14 Hz, 1H), 6.8 – 6.7 (m, 1H), 6.6 (t, *J* = 2.70 Hz, 1H), 5.9 (d, *J* = 38.81 Hz, 1H), 4.2 – 4.1 (m, 1H), 4.1 – 4.0 (m, 1H), 3.8 (d, *J* = 1.89 Hz, 3H), 3.0 – 2.8 (m, 2H), 2.7 – 2.6 (m, 1H), 2.5 – 2.4 (m, 3H), 2.3 – 2.1 (m, 2H), 2.0 – 1.9 (m, 1H), 1.9 – 1.8 (m, 5H), 1.8 – 1.6 (m, 2H), 1.2 – 1.1 (m, 3H).

^{13}**C NMR** (126 MHz, Chloroform-*d*) δ 200.8, 200.4, 198.6, 198.5, 173.3, 173.0, 163.3, 163.3, 154.4, 154.2, 146.3, 146.3, 129.9, 129.8, 128.7, 127.9, 126.1, 126.1, 113.1, 113.0, 112.3, 112.3, 63.4, 63.3, 61.2, 61.2, 55.4, 55.4, 44.0, 44.0, 36.6, 36.6, 36.3, 36.0, 32.5, 32.0, 31.3, 30.8, 29.5, 29.4, 26.7, 26.3, 24.3, 23.2, 14.0, 14.0

HRMS (ESI): $C_{23}H_{28}O_5Na$ $[M+Na]^+$ calculated: 407.1834, found 407.1836.

Isopropyl 1-((6-methoxy-1-oxo-1,2,3,4-tetrahydronaphthalen-2-yl)methyl)-4-methyl-2-oxocyclohept-3-ene-1-carboxylate(3c)

^{1}**H NMR** (500 MHz, Chloroform-*d*) δ 7.9 (dd, *J* = 8.73, 5.80 Hz, 1H), 6.8 – 6.7 (m, 1H), 6.6 (s, 1H), 5.9 (d, *J* = 39.66 Hz, 1H), 5.1 – 4.9 (m, 1H), 3.8 (d, *J* = 2.07 Hz, 3H), 3.0 – 2.8 (m, 2H), 2.7 (dd, *J* = 14.24, 5.01 Hz, 1H), 2.6 – 2.4 (m, 3H), 2.3 – 2.1 (m, 2H), 2.0 – 1.9 (m, 1H), 1.9 – 1.8 (m, 5H), 1.8 – 1.6 (m, 2H), 1.2 – 1.1 (m, 6H).

^{13}**C NMR** (126 MHz, Chloroform-*d*) δ 200.8, 200.4, 198.6, 198.4, 172.8, 172.5, 163.3, 163.3, 154.3, 154.0, 146.3, 146.3, 129.9, 128.9, 128.2, 126.1, 126.1, 113.1, 113.0, 112.3, 112.3, 68.7, 63.4, 63.4, 55.4, 44.0, 44.0, 36.5, 36.4, 36.3, 35.8, 32.5, 32.1, 31.3, 30.9, 29.5, 29.5, 26.7, 26.2, 24.6, 23.4, 21.6, 21.5, 21.5, 21.4.

HRMS (ESI): $C_{24}H_{30}O_5Na$ $[M+Na]^+$ calculated: 421.1911, found 421.1911.

Tert-butyl 1-((6-methoxy-1-oxo-1,2,3,4-tetrahydronaphthalen-2-yl)methyl)-4-methyl-2-oxocyclohept-3-ene-1-carboxylate(3d)

^{1}**H NMR** (500 MHz, Chloroform-*d*) δ 7.9 (dd, *J* = 8.70, 4.98 Hz, 1H), 6.8 – 6.7 (m, 1H), 6.6 (s, 1H), 5.9 (d, *J* = 34.74 Hz, 1H), 3.8 (s, 3H), 3.0 – 2.9 (m, 2H), 2.7 – 2.6 (m, 1H), 2.5 – 2.4 (m, 3H), 2.3 – 2.1 (m, 2H), 2.0 – 1.7 (m, 7H), 1.5 – 1.4 (m, 1H), 1.4 (d, *J* = 13.18 Hz, 9H).

^{13}**C NMR** (126 MHz, Chloroform-*d*) δ 200.9, 200.5, 198.6, 198.4, 172.3, 172.1, 163.3, 163.2, 154.1, 153.8, 146.3, 146.3, 129.9, 129.0, 128.3, 126.2, 113.1, 113.0, 112.4, 112.3, 81.6, 64.0 (d, *J* = 2.17 Hz), 55.4, 44.3, 43.9, 36.3, 36.3, 36.0, 35.8, 32.4, 31.9, 31.3, 30.9, 29.5, 29.4, 27.8, 27.8, 26.7, 26.2, 24.7, 23.6.

HRMS (ESI): $C_{25}H_{32}O_5Na$ $[M+Na]^+$ calculated: 435.2147, found 435.2142.

Isobutyl 1-((6-methoxy-1-oxo-1,2,3,4-tetrahydronaphthalen-2-yl)methyl)-4-methyl-2-oxocyclohept-3-ene-1-carboxylate(3e)

^{1}H NMR (500 MHz, Chloroform-*d*) δ 7.9 (dd, *J* = 8.75, 4.39 Hz, 1H), 6.8 (ddd, *J* = 8.81, 4.65, 2.58 Hz, 1H), 6.6 (t, *J* = 2.73 Hz, 1H), 5.9 (d, *J* = 38.11 Hz, 1H), 3.9 – 3.9 (m, 1H), 3.9 – 3.7 (m, 4H), 3.0 – 2.8 (m, 2H), 2.8 – 2.6 (m, 1H), 2.6 – 2.4 (m, 3H), 2.3 – 2.1 (m, 2H), 2.0 – 1.7 (m, 9H), 0.9 (d, *J* = 6.76 Hz, 3H), 0.9 (dd, *J* = 6.71, 3.21 Hz, 3H).

^{13}C NMR (126 MHz, Chloroform-*d*) δ 200.6, 200.3, 198.6, 198.5, 173.4, 173.2, 163.3, 163.3, 154.6, 154.2, 146.3, 146.3, 129.9, 129.9, 128.8, 128.1, 126.1, 126.1, 113.1, 113.0, 112.3, 112.3, 71.6, 71.4, 63.4, 55.4, 44.0, 36.7, 36.6, 36.5, 36.0, 32.6, 32.1, 31.3, 30.9, 29.5, 29.5, 27.6, 27.6, 26.8, 26.4, 24.4, 23.3, 19.1, 19.1.

HRMS (ESI): $C_{25}H_{32}O_5Na$ $[M+Na]^+$ calculated: 435.2147, found 435.2149.

Benzyl 1-((6-methoxy-1-oxo-1,2,3,4-tetrahydronaphthalen-2-yl)methyl)-4-methyl-2-oxocyclohept-3-ene-1-carboxylate(3f)

^{1}H NMR (500 MHz, Chloroform-*d*) δ 7.9 (dd, *J* = 8.71, 2.64 Hz, 1H), 7.4 – 7.3 (m, 5H), 6.8 – 6.7 (m, 1H), 6.6 (t, *J* = 3.32 Hz, 1H), 5.8 (d, *J* = 42.21 Hz, 1H), 5.2 (dd, *J* = 24.74, 12.19 Hz, 1H), 5.0 (dd, *J* = 22.10, 12.22 Hz, 1H), 3.8 (d, *J* = 1.93 Hz, 4H), 3.0 – 2.6 (m, 3H), 2.6 – 2.4 (m, 2H), 2.4 – 2.3 (m, 1H), 2.3 – 2.0 (m, 2H), 2.0 – 1.6 (m, 8H).

^{13}C NMR (126 MHz, Chloroform-*d*) δ 200.5, 200.1, 198.7, 198.5, 173.1, 172.8, 163.3, 163.3, 154.8, 154.4, 129.9, 129.9, 128.7, 128.5, 128.5, 128.3, 128.1, 128.0, 126.1, 113.1, 113.0, 112.3, 67.1, 67.0, 63.5, 63.4, 55.4, 55.4, 44.0, 43.9, 36.8, 36.5, 36.4, 35.8, 32.6, 32.1, 31.3, 30.9, 29.5, 29.4, 26.7, 26.3, 24.4, 23.4.

HRMS (ESI): $C_{28}H_{31}O_5Na$ $[M+Na]^+$ calculated: 447.2171, found 447.2172.

2. General Procedure for Synthesis of Compound 2

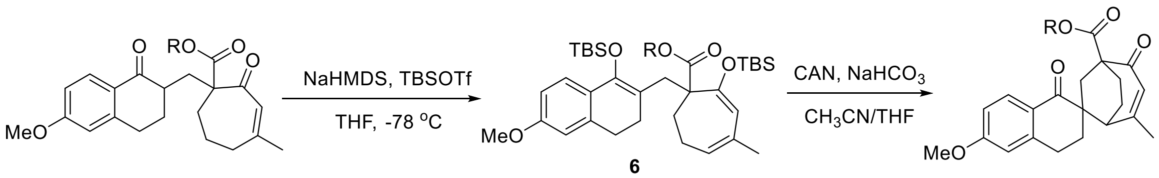

3 6 2

To a solution of compound **3** (0.40 mmol, 1.0 equiv.) in THF (4 mL) was added NaHMDS (2M in THF, 0.60 mL, 1.60 mmol, 4.0 equiv.) dropwise at -78°C. After stirring for 1 hour at -78 °C, TBSOTf (0.23 mL, 1.00 mmol, 2.5 equiv.) was added and the mixture was stirred for another 1 hour at -78 °C. Aq. sat $NaHCO_3$ (2 mL) and water was added, the aqueous layer extracted with EtOAc (3 × 5 mL). The yellow combined layers were washed with brine (3 × 3 mL), dried over Na_2SO_4 and concentrated in vacuo. Further purification by Alkaline Alumina (PE : EtOAc, 80:1) to obtain corresponding enol silyl ether **6**.

CAN(2.0 equiv.) and $NaHCO_3$(4.0 equiv.) in CH_3CN was stirred for 10 minutes at 0°C, and enol silyl ether (1 equiv.) in CH_3CN (THF for solubilization) was added dropwise. After stirring for 30 minutes to 1 hour, the enol silyl ether was consumed. The 1N HCl was added and the aqueous layer extracted with EtOAc(3 x 5 mL). The combined organic layers were washed with brine (3 × 3 mL mL), dried over Na_2SO_4 and concentrated in vacuo. Flash chromatography (PE : EtOAc, 5:1) furnished product **(2′)** as a white solid.

Methyl 6'-methoxy-4-methyl-1',2-dioxo-3',4'-dihydro-1'H-spiro[bicyclo[3.2.2]nonane-6,2'-naphthalen]-3-ene-1-carboxylate(2a′)

^{1}H NMR (500 MHz, Chloroform-*d*) δ 8.0 (d, J = 8.79 Hz, 1H), 6.8 (dd, J = 8.79, 2.46 Hz, 1H), 6.7 (d, J = 2.50 Hz, 1H), 6.1 (s, 1H), 3.8 (s, 3H), 3.7 (s, 3H), 3.1 – 3.0 (m, 1H), 2.9 – 2.8 (m, 2H), 2.7 (dt, J = 5.18, 1.76 Hz, 1H), 2.2 – 2.1 (m, 3H), 2.1 – 1.9 (m, 3H), 1.9 – 1.8 (m, 1H), 1.6 – 1.5 (m, 2H).

^{13}C NMR (126 MHz, Chloroform-*d*) δ 200.9, 198.9, 173.1, 163.7, 163.0, 144.7, 131.0, 129.4, 124.7, 113.8, 112.2, 59.9, 55.5, 52.2, 48.4, 42.7, 34.9, 33.5, 27.5, 26.2, 23.0, 22.7.

HRMS (ESI): $C_{22}H_{24}O_5Na$ $[M+Na]^+$ calculated: 391.1521, found 391.1519.

Ethyl 6'-methoxy-4-methyl-1',2-dioxo-3',4'-dihydro-1'H-spiro[bicyclo[3.2.2]nonane-6,2'-naphthalen]-3-ene-1-carboxylate(2b′)

^{1}H NMR (500 MHz, Chloroform-*d*) δ 8.0 (d, *J* = 8.78 Hz, 1H), 6.8 (dd, *J* = 8.83, 2.57 Hz, 1H), 6.7 (s, 1H), 6.1 (s, 1H), 4.2 (q, 2H), 3.8 (s, 3H), 3.1 – 3.0 (m, 1H), 2.9 – 2.8 (m, 2H), 2.7 (d, *J* = 4.52 Hz, 2H), 2.2 – 2.1 (m, 4H), 2.1 – 1.9 (m, 3H), 1.9 – 1.8 (m, 1H), 1.7 – 1.5 (m, 2H), 1.3 (t, *J* = 7.12 Hz, 4H).

^{13}C NMR (126 MHz, Chloroform-*d*) δ 201.1, 198.9, 172.7, 163.7, 162.8, 144.8, 131.0, 129.5, 124.7, 113.8, 112.2, 61.0, 59.7, 55.5, 48.5, 42.7, 34.9, 33.5, 27.5, 26.2, 23.0, 22.6, 14.1.

HRMS (ESI): $C_{23}H_{26}O_5Na$ $[M+Na]^+$ calculated: 405.1678, found 405.1679.

Isopropyl 6'-methoxy-4-methyl-1',2-dioxo-3',4'-dihydro-1'H-spiro[bicyclo[3.2.2]nonane-6,2'-naphthalen]-3-ene-1-carboxylate(2c′)

^{1}H NMR (500 MHz, Chloroform-*d*) δ 8.0 (d, *J* = 8.77 Hz, 1H), 6.8 (d, *J* = 8.77 Hz, 1H), 6.6 (s, 1H), 6.1 (s, 1H), 5.1 – 5.0 (m, 1H), 3.8 (s, 2H), 3.1 – 3.0 (m, 1H), 2.8 (t, *J* = 16.07 Hz, 3H), 2.7 (d, *J* = 3.93 Hz, 2H), 2.2 – 2.1 (m, 6H), 2.0 – 2.0 (m, 2H), 1.9 (ddp, *J* = 13.98, 9.18, 2.30 Hz, 1H), 1.9 – 1.7 (m, 1H), 1.6 (d, *J* = 13.57 Hz, 2H), 1.2 (d, *J* = 6.27 Hz, 9H).

^{13}C NMR (126 MHz, Chloroform-*d*) δ 201.1, 198.9, 172.1, 163.6, 162.5, 144.8, 130.9, 129.5, 124.7, 113.8, 112.2, 68.2, 59.5, 55.5, 48.5, 42.7, 34.9, 33.5, 27.5, 26.3, 23.1, 22.6, 21.7, 21.7.

HRMS (ESI): $C_{24}H_{28}O_5Na$ $[M+Na]^+$ calculated: 419.1834, found 419.1838.

2c: ^{1}H NMR (500 MHz, Chloroform-*d*) δ 8.0 (d, *J* = 8.77 Hz, 1H), 6.8 (dd, *J* = 8.78, 2.48 Hz, 1H), 6.7 (d, *J* = 2.31 Hz, 1H), 6.0 (s, 1H), 5.1 – 5.0 (m, 1H), 3.9 (s, 3H), 3.2 (dd, *J* = 14.97, 1.97 Hz, 1H), 3.2 – 3.0 (m, 1H), 3.0 – 2.9 (m, 1H), 2.7 (d, *J* = 5.70 Hz, 1H), 2.3 – 2.2 (m, 1H), 2.2 – 2.1 (m, 2H), 2.1 – 2.0 (m, 2H), 1.8 – 1.7 (m, 1H), 1.6 (d, *J* = 1.21 Hz, 3H), 1.4 (d, *J* = 15.05 Hz, 1H), 1.3 (dd, *J* = 10.41, 6.28 Hz, 6H).

^{13}C NMR (126 MHz, Chloroform-*d*) δ 200.1, 197.5, 172.4, 163.5, 163.3, 130.7, 128.9, 125.3, 113.8, 112.4, 68.4, 59.1, 55.5, 47.4, 42.0, 34.8, 32.8, 27.3, 26.2, 23.7, 23.0, 21.8, 21.6.

Tert-butyl 6'-methoxy-4-methyl-1',2-dioxo-3',4'-dihydro-1'H-spiro[bicyclo[3.2.2]nonane-6,2'-naphthalen]-3-ene-1-carboxylate(2d′)

^{1}H NMR (500 MHz, Chloroform-*d*) δ 8.0 (d, *J* = 8.73 Hz, 1H), 6.9 (dd, *J* = 8.82, 2.54 Hz, 1H), 6.7 (s, 1H), 6.1 (s, 1H), 3.9 (s, 3H), 3.1 – 3.0 (m, 1H), 2.9 – 2.8 (m, 2H), 2.7 – 2.7 (m, 1H), 2.2 – 2.1 (m, 4H), 2.1 – 2.0 (m, 2H), 2.0 – 1.9 (m, 1H), 1.9 – 1.8 (m, 1H), 1.6 – 1.5 (m, 2H), 1.5 (s, 9H).

^{13}C NMR (126 MHz, Chloroform-*d*) δ 201.6, 198.9, 171.8, 163.6, 162.0, 144.8, 131.0, 129.6, 124.7, 113.7, 112.2, 80.9, 59.9, 55.5, 48.6, 42.7, 34.9, 33.6, 28.0, 27.4, 26.3, 23.3, 22.4.

HRMS (ESI): $C_{25}H_{30}O_5Na$ $[M+Na]^+$ calculated: 433.1991, found 419.1990.

Isobutyl 6'-methoxy-4-methyl-1',2-dioxo-3',4'-dihydro-1'H-spiro[bicyclo[3.2.2]nonane-6,2'-naphthalen]-3-ene-1-carboxylate(2e′)

^{1}H NMR (500 MHz, Chloroform-*d*) δ 8.0 (d, *J* = 8.82 Hz, 1H), 6.8 (d, *J* = 8.77 Hz, 1H), 6.6 (s, 1H), 6.1 (s, 1H), 4.0 – 3.9 (m, 2H), 3.8 (s, 2H), 3.1 – 3.0 (m, 1H), 2.9 (d, *J* = 14.56 Hz, 1H), 2.7 (d, *J* = 4.30 Hz, 1H), 2.2 – 2.2 (m, 1H), 2.1 (s, 0H), 2.1 – 1.9 (m, 4H), 1.9 – 1.7 (m, 1H), 1.6 (d, *J* = 14.64 Hz, 2H), 0.9 (d, *J* = 6.76 Hz, 6H).

^{13}C NMR (126 MHz, Chloroform-*d*) δ 200.9, 198.9, 172.7, 163.7, 162.8, 144.8, 130.9, 129.4, 124.6, 113.8, 112.2, 71.0, 59.9, 55.5, 48.5, 42.6, 34.9, 33.5, 27.7, 27.5, 26.2, 23.0, 22.6, 19.1.

HRMS (ESI): $C_{25}H_{30}O_5Na$ $[M+Na]^+$ calculated: 433.1991, found 419.1993.

X-Ray Crystallographic Data

The single crystal of **3c** and **2a′** which were used for the determination of its relative configurations via X-ray crystallography (see below). The intensity data were collected using graphite-monochromated Mo Kα radiation.

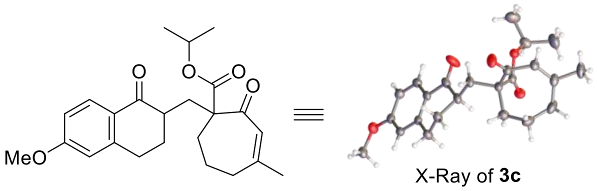

3c

Identification code	3c
Empirical formula	$C_{24}H_{30}O_5$
Formula weight	398.48
Temperature/K	219.99(10)
Crystal system	monoclinic
Space group	$P2_1/n$
a/Å	7.5638(2)
b/Å	14.1378(3)
c/Å	20.3678(4)
α/°	90
β/°	99.070(2)
γ/°	90
Volume/Å^3	2150.81(9)
Z	4
ρ_{calc}g/cm^3	1.231
μ/mm^{-1}	0.688
F(000)	856.0
Crystal size/mm^3	0.14 × 0.13 × 0.12
Radiation	Cu Kα (λ = 1.54184)
2Θ range for data collection/°	7.644 to 146.676
Index ranges	$-7 \leq h \leq 9, -17 \leq k \leq 17, -25 \leq l \leq 24$
Reflections collected	8447
Independent reflections	4208 [R_{int} = 0.0218, R_{sigma} = 0.0233]
Data/restraints/parameters	4208/0/267
Goodness-of-fit on F^2	1.031
Final R indexes [I>=2σ (I)]	R_1 = 0.0509, wR_2 = 0.1372
Final R indexes [all data]	R_1 = 0.0567, wR_2 = 0.1415
Largest diff. peak/hole / e Å^{-3}	0.22/-0.17

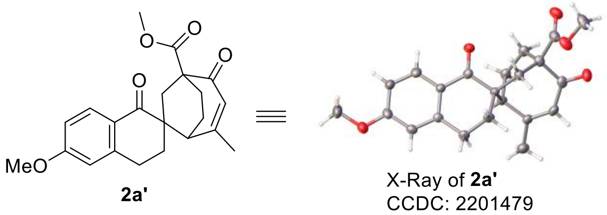

2a'

Identification code	**2a′**
Empirical formula	$C_{22}H_{24}O_5$
Formula weight	368.41
Temperature/K	170.0
Crystal system	triclinic
Space group	P-1
a/Å	10.220(4)
b/Å	12.429(7)
c/Å	15.708(10)
α/°	96.530(19)
β/°	90.983(15)
γ/°	111.540(15)
Volume/Å^3	1840.2(17)
Z	4
ρ_{calc}g/cm^3	1.330
μ/mm^{-1}	0.094
F(000)	784.0
Crystal size/mm^3	0.12 × 0.05 × 0.02
Radiation	MoKα (λ = 0.71073)
2Θ range for data collection/°	4.13 to 50.696
Index ranges	-11 ≤ h ≤ 12, -14 ≤ k ≤ 14, -18 ≤ l ≤ 18
Reflections collected	19032
Independent reflections	6714 [R_{int} = 0.1453, R_{sigma} = 0.1775]
Data/restraints/parameters	6714/0/493
Goodness-of-fit on F^2	1.004
Final R indexes [I>=2σ (I)]	R_1 = 0.0944, wR_2 = 0.2051
Final R indexes [all data]	R_1 = 0.2137, wR_2 = 0.2753
Largest diff. peak/hole / e Å^{-3}	0.40/-0.34

References

[1] (a) Collier S J. Carboxylic Acid Esters: Synthesis from Carbonic Acid Derivatives[J]. *ChemInform*, **2008**, *39(49)*.

(b) Liao Z, Zhang J, Cao T, et al. Copper-Catalyzed Asymmetric Synthesis of Bicyclo [3. n. 1] alkenones[J]. *The Journal of Organic Chemistry*, **2021**, *86(7)*: 5388-5400.

[2] Li Y P, Li Z Q, Zhou B, et al. Chiral Spiro Phosphoric Acid-Catalyzed Friedel–Crafts Conjugate Addition/Enantioselective Protonation Reactions[J]. *ACS Catalysis*, **2019**, *9(7)*: 6522-6529.

NMR Spectra

1. NMR Spectra for Compounds of **3a-3f**

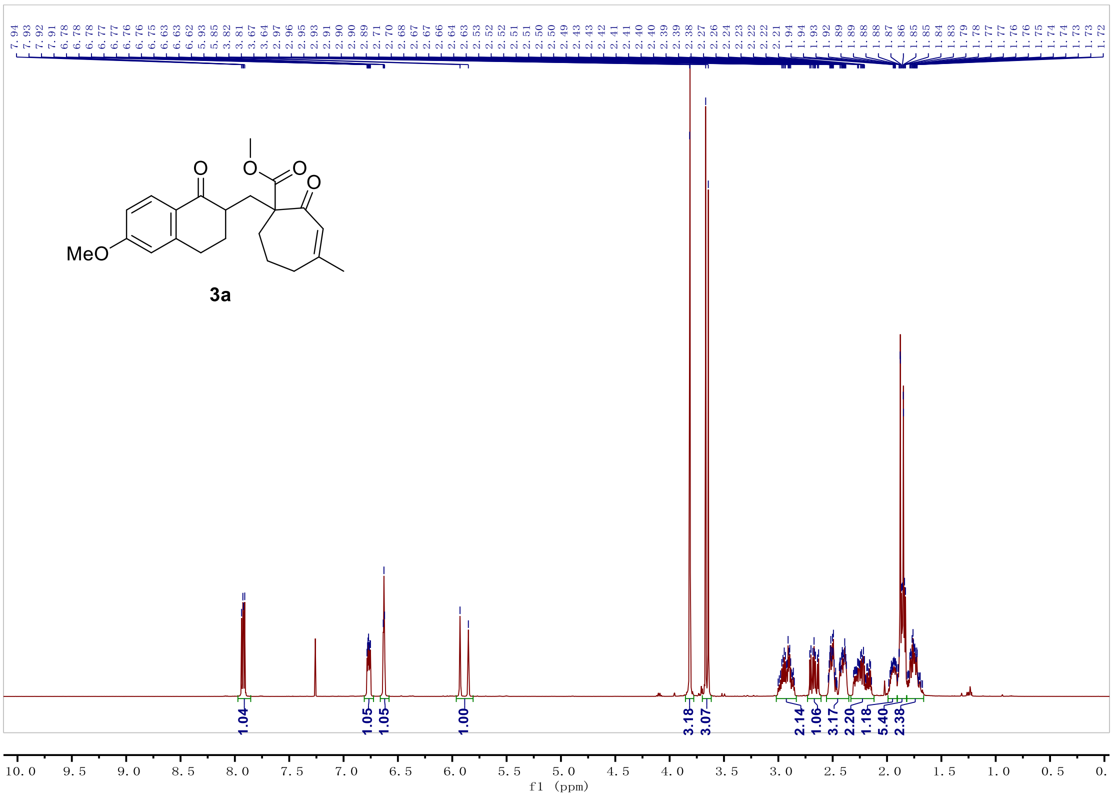

^{1}H NMR (500 MHz, CDCl3) spectrum of **3a**

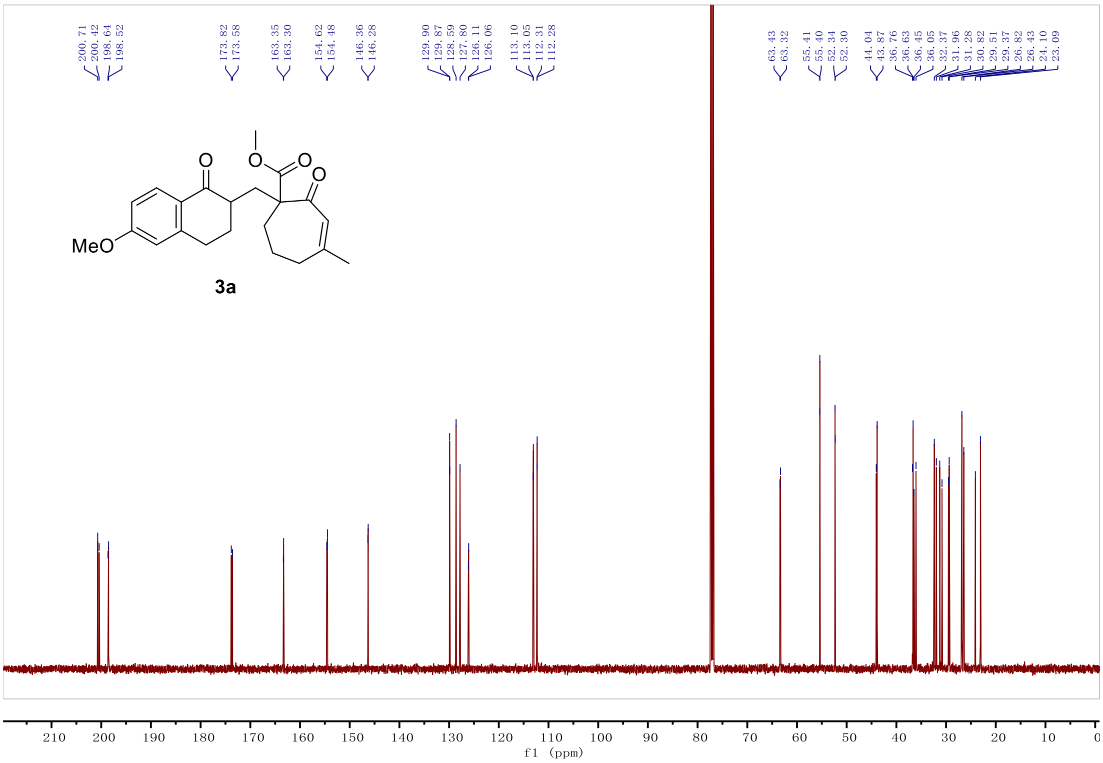

^{13}C NMR (126 MHz, CDCl3) spectrum of **3a**

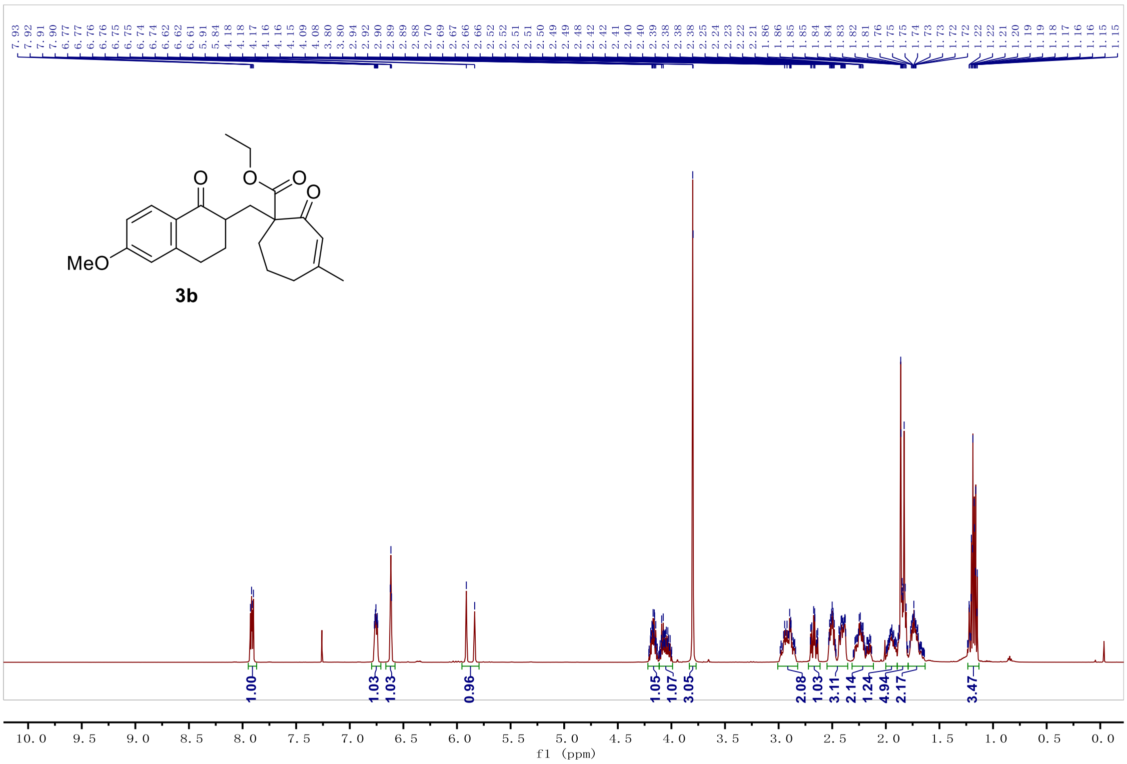

^{1}H NMR (500 MHz, CDCl3) spectrum of **3b**

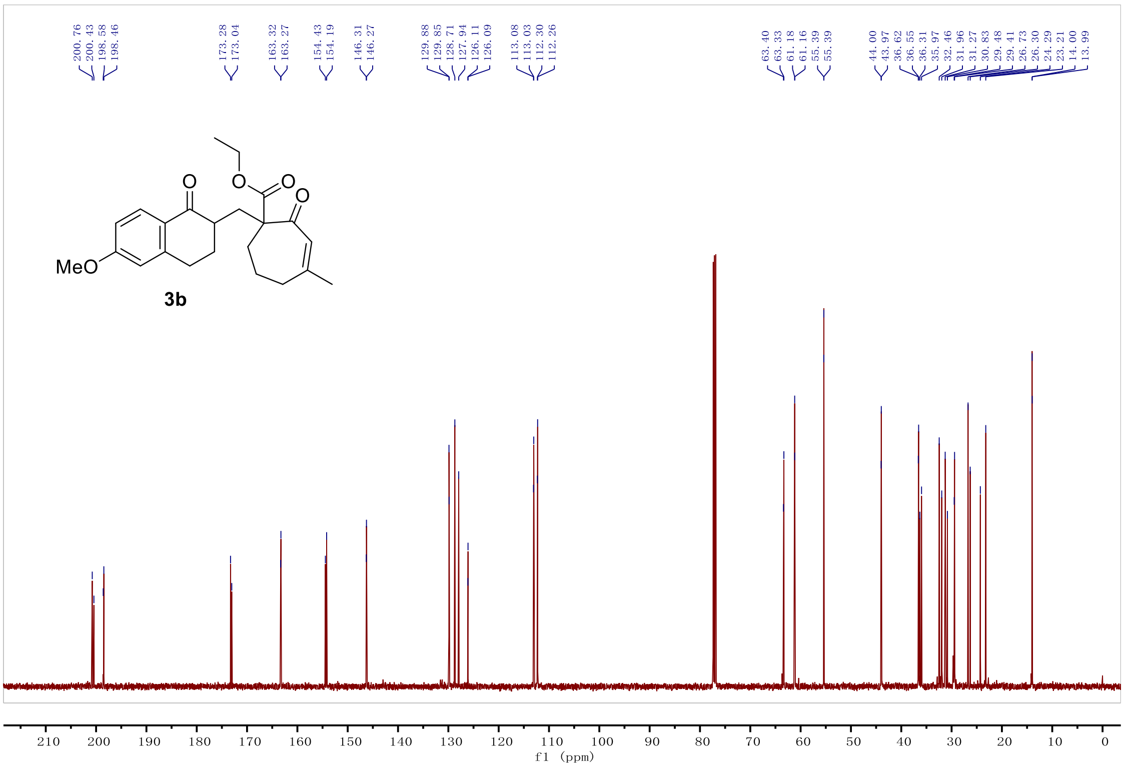

^{13}C NMR (126 MHz, CDCl3) spectrum of **3b**

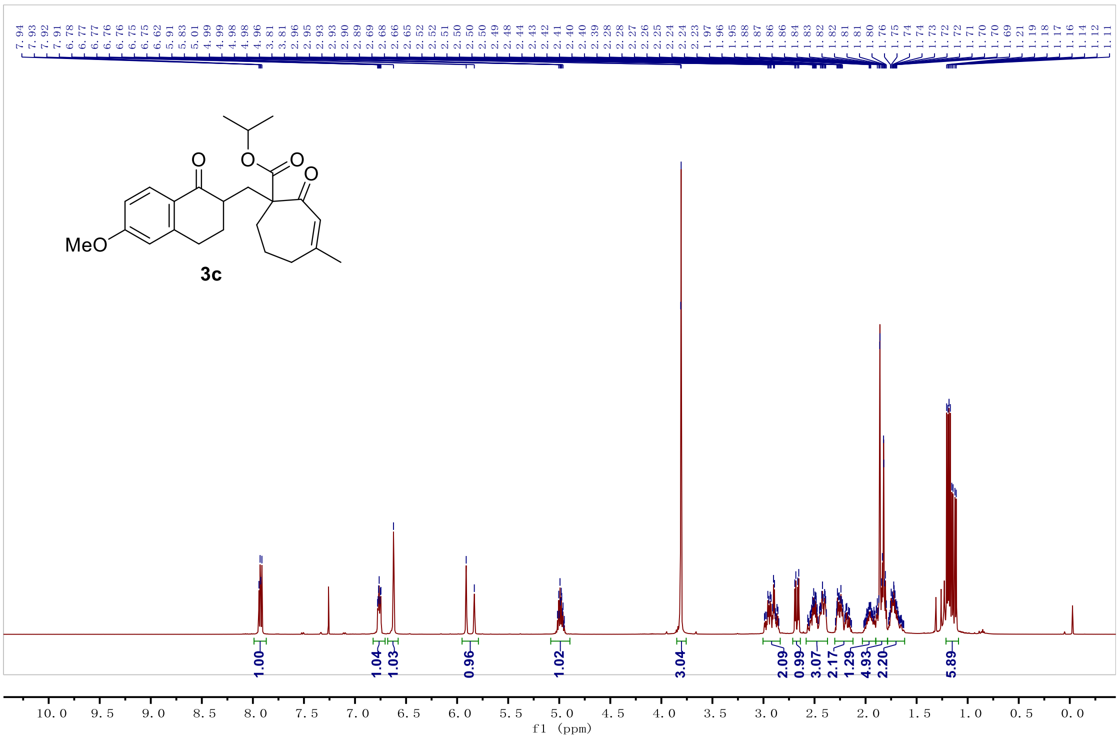

^{1}H NMR (500 MHz, CDCl3) spectrum of **3c**

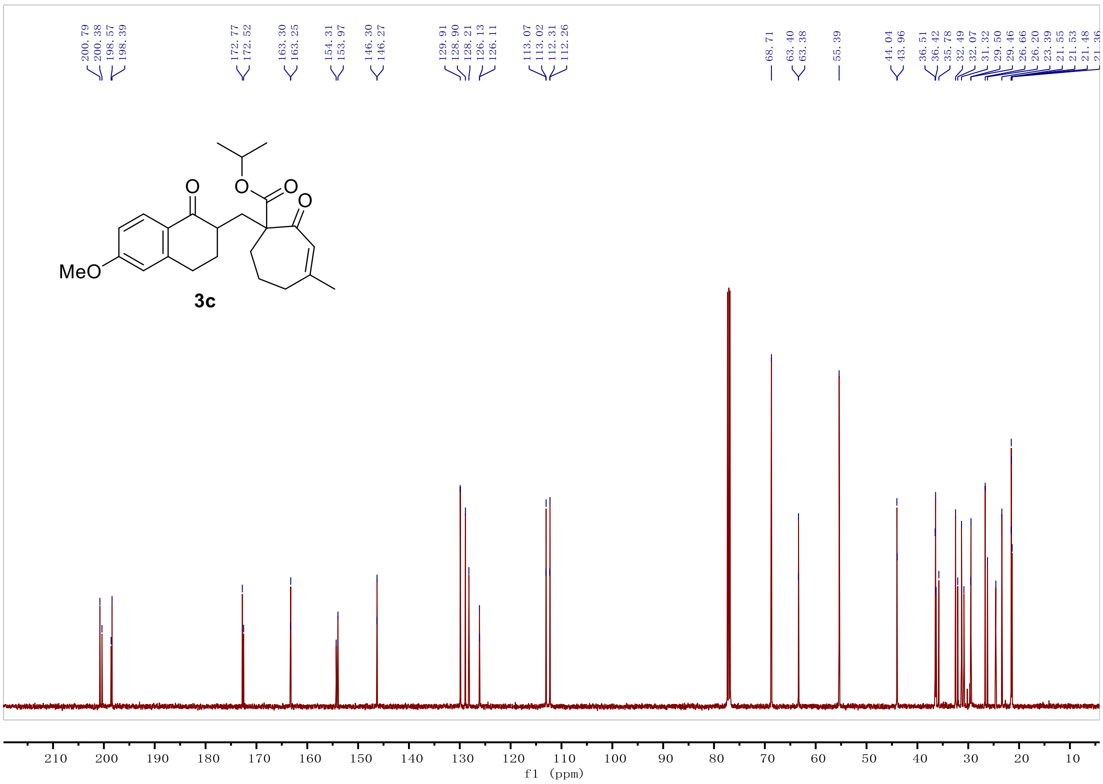

^{13}C NMR (126 MHz, CDCl3) spectrum of **3c**

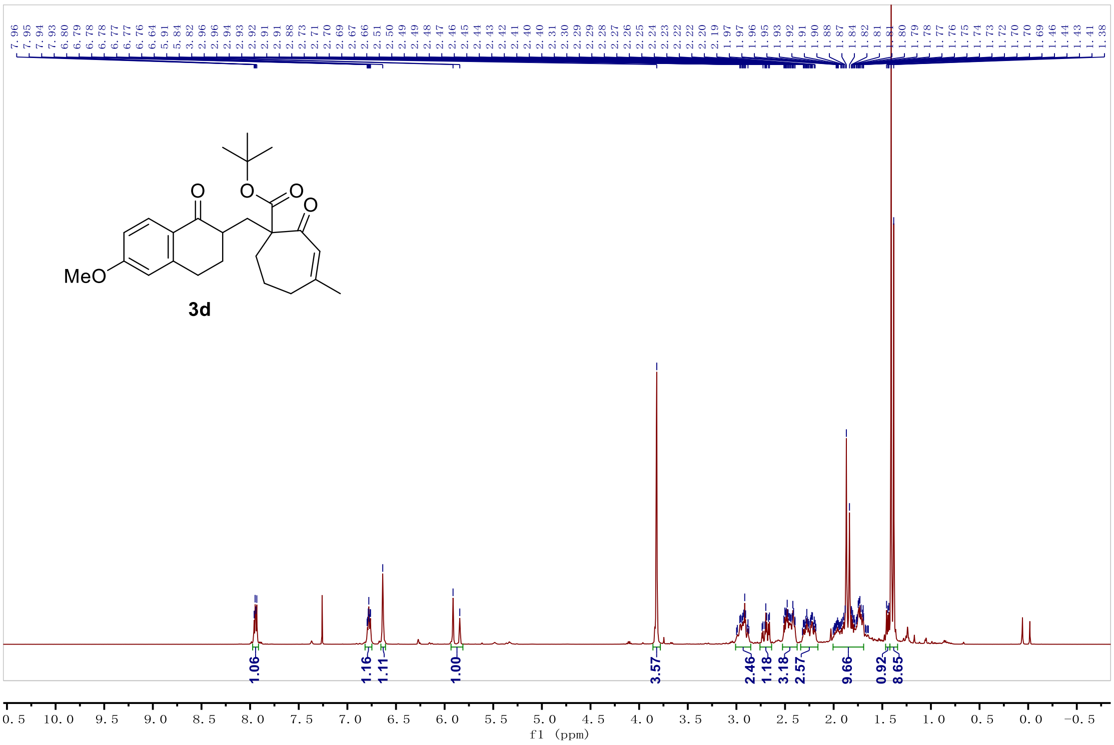

^{1}H NMR (500 MHz, CDCl3) spectrum of **3d**

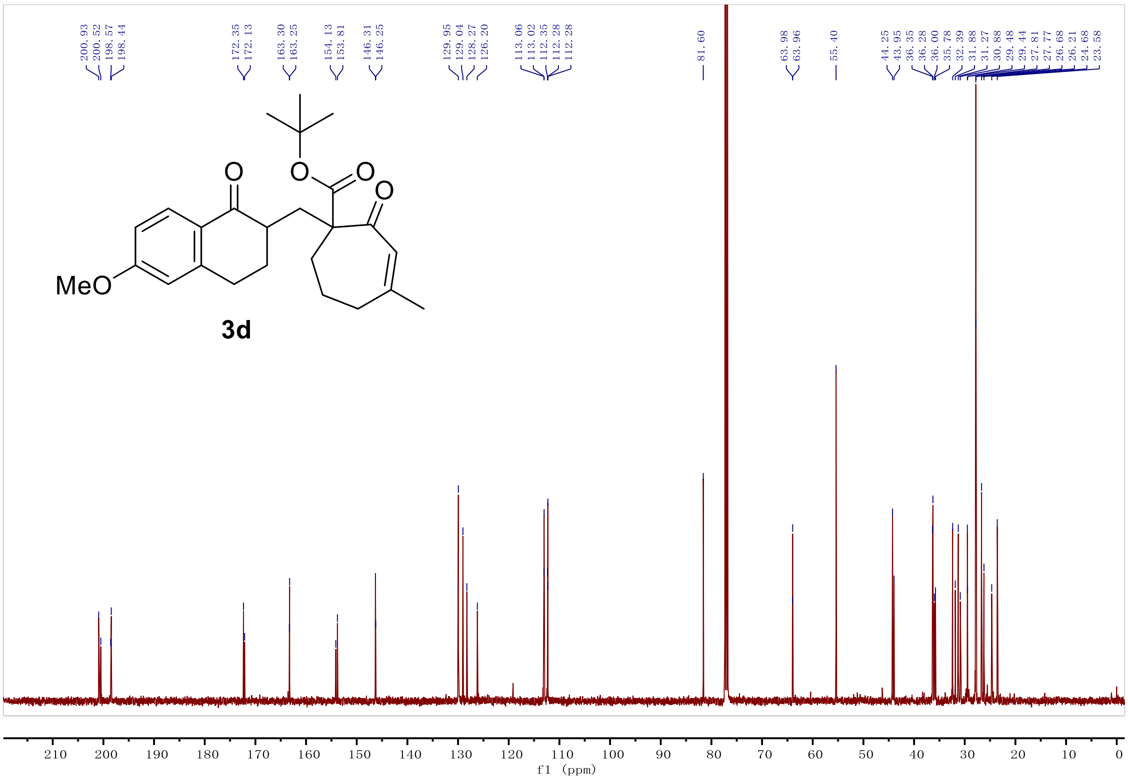

^{13}C NMR (126 MHz, CDCl3) spectrum of **3d**

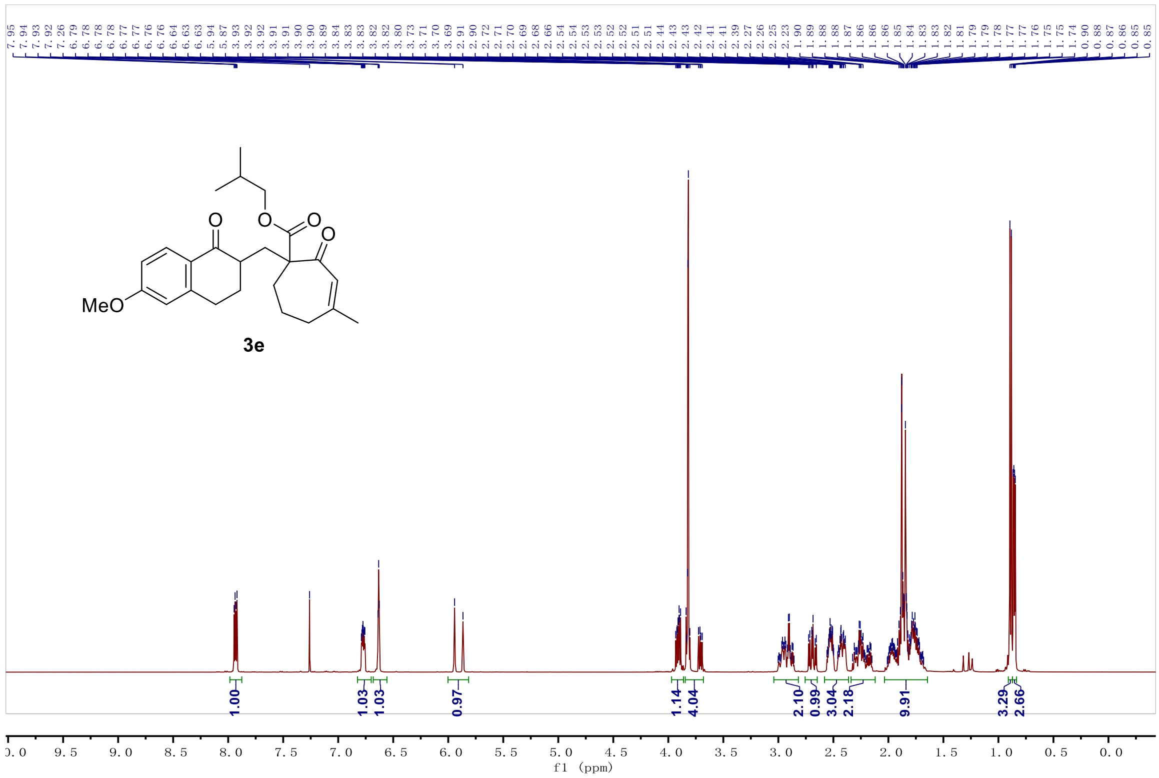

^{1}H NMR (500 MHz, CDCl3) spectrum of **3e**

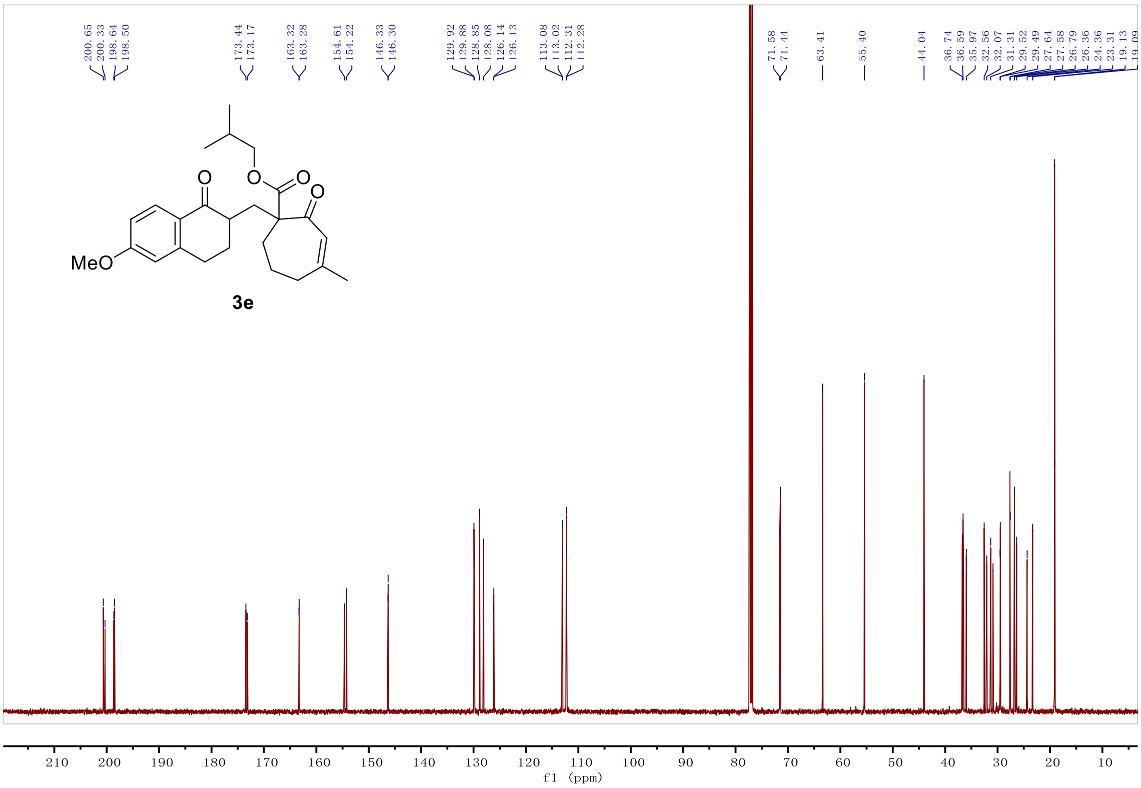

^{13}C NMR (126 MHz, CDCl3) spectrum of **3e**

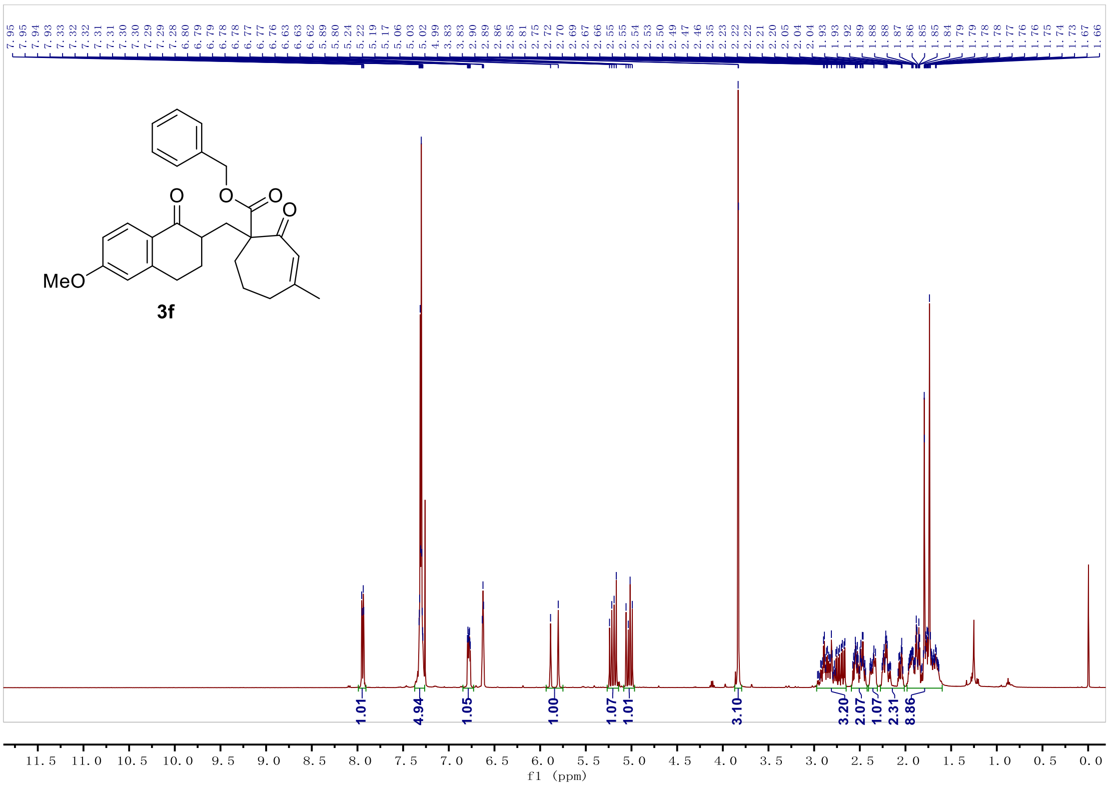

^{1}H NMR (500 MHz, CDCl3) spectrum of **3f**

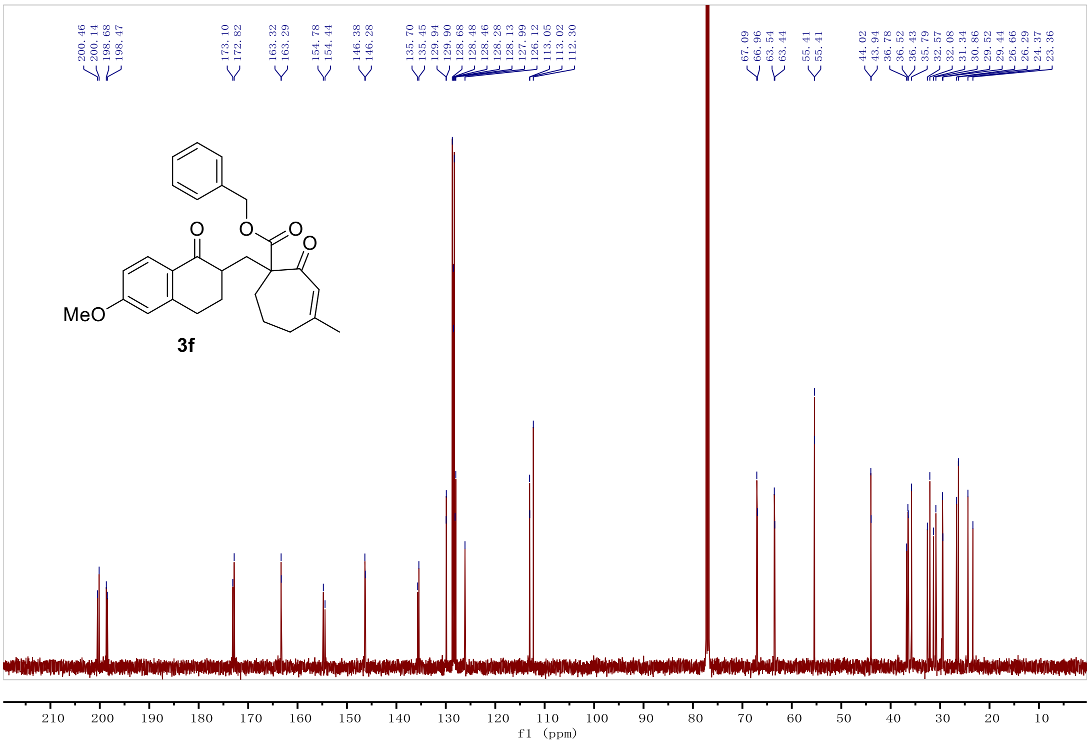

^{13}C NMR (126 MHz, CDCl3) spectrum of **3f**

2. NMR Spectra for Compounds of **2a′-2e′**

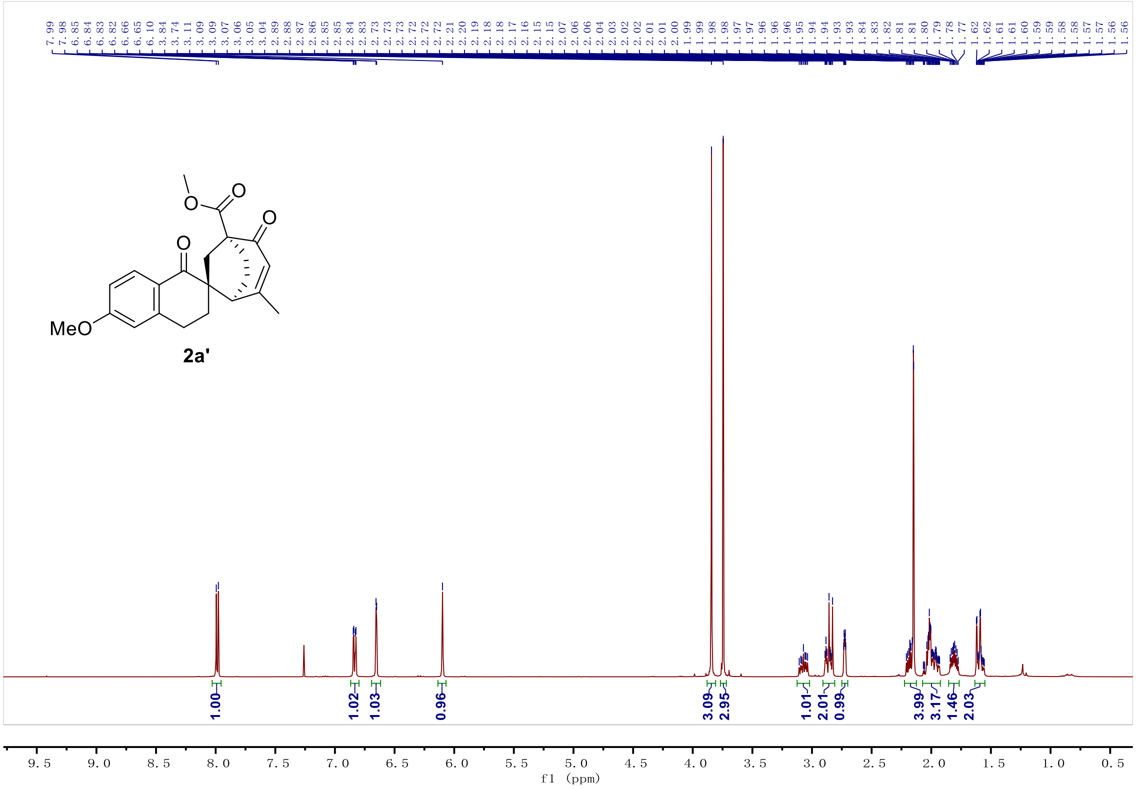

^{1}H NMR (500 MHz, CDCl3) spectrum of **2a′**

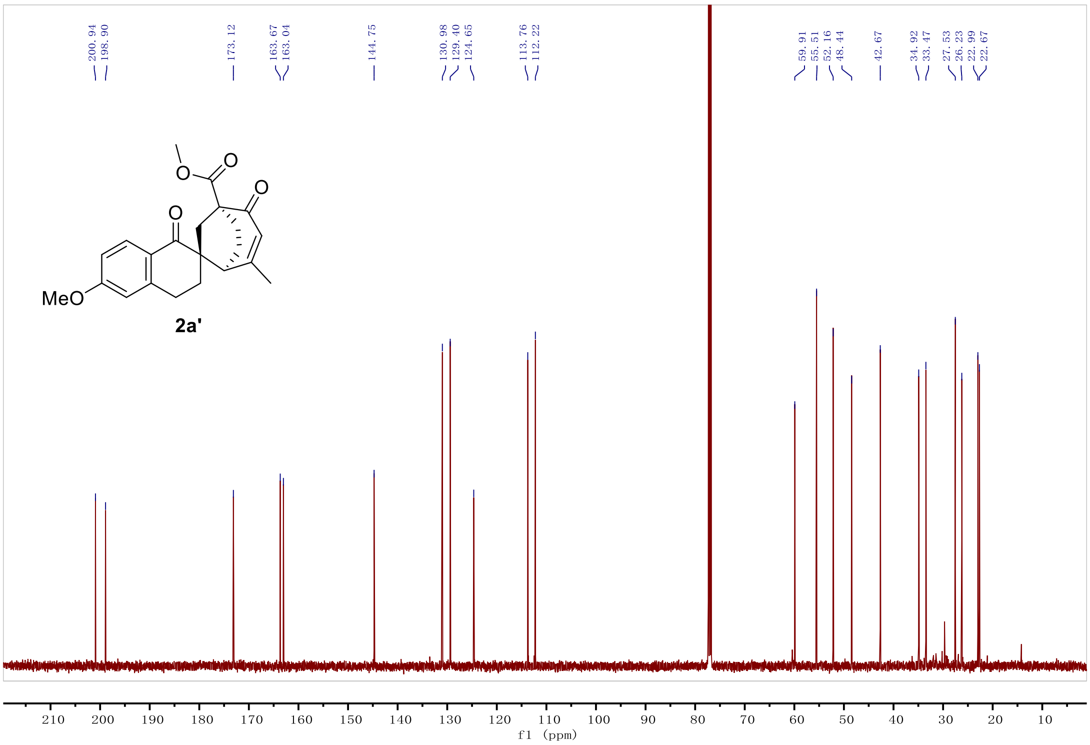

^{13}C NMR (126 MHz, CDCl3) spectrum of **2a′**

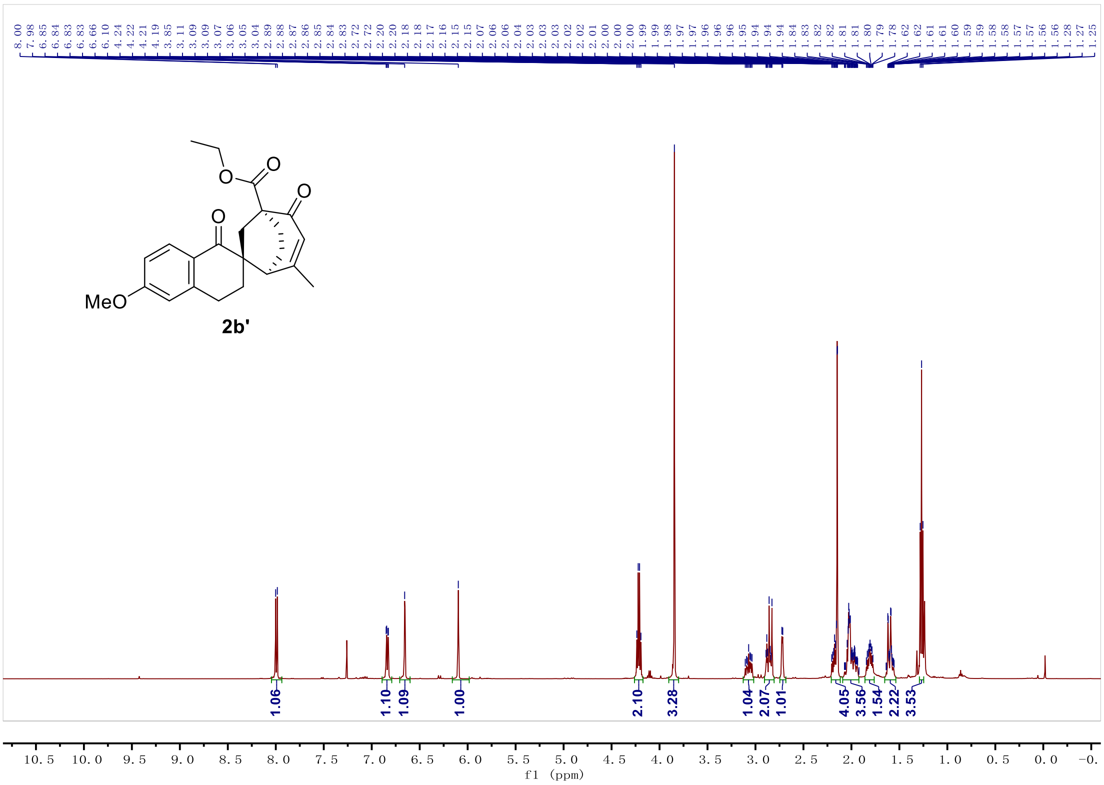

^{1}H NMR (500 MHz, CDCl3) spectrum of **2b′**

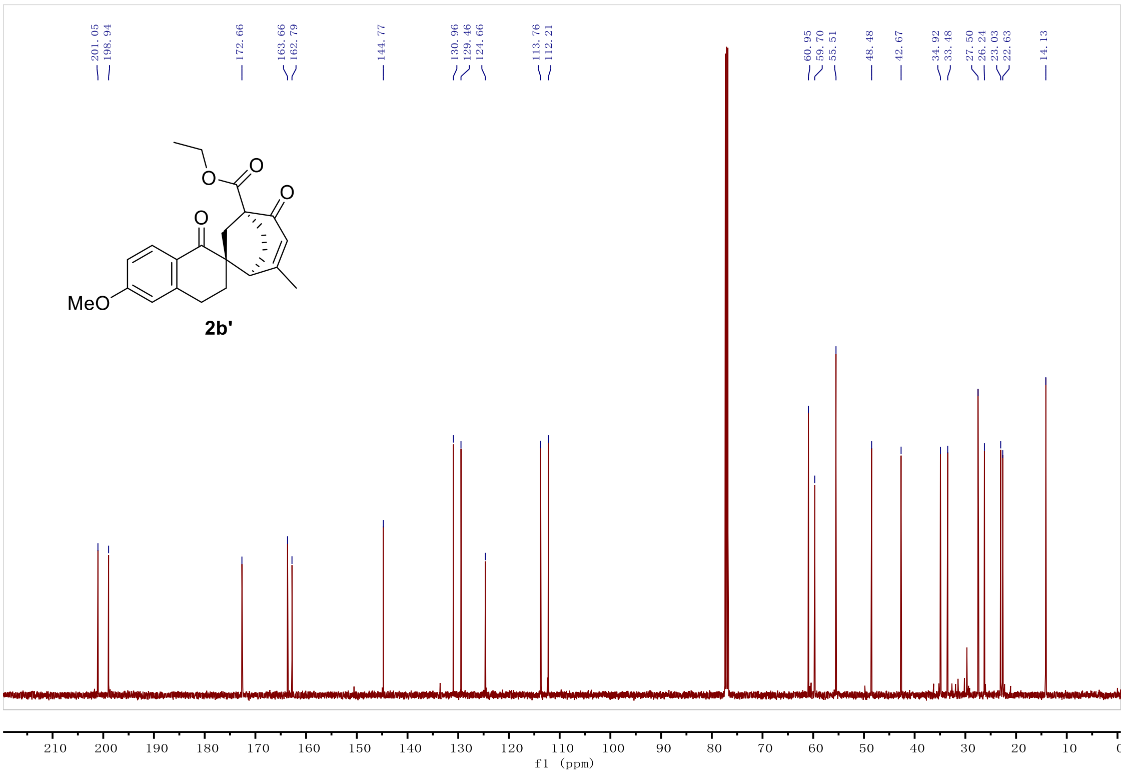

^{13}C NMR (126 MHz, CDCl3) spectrum of **2b′**

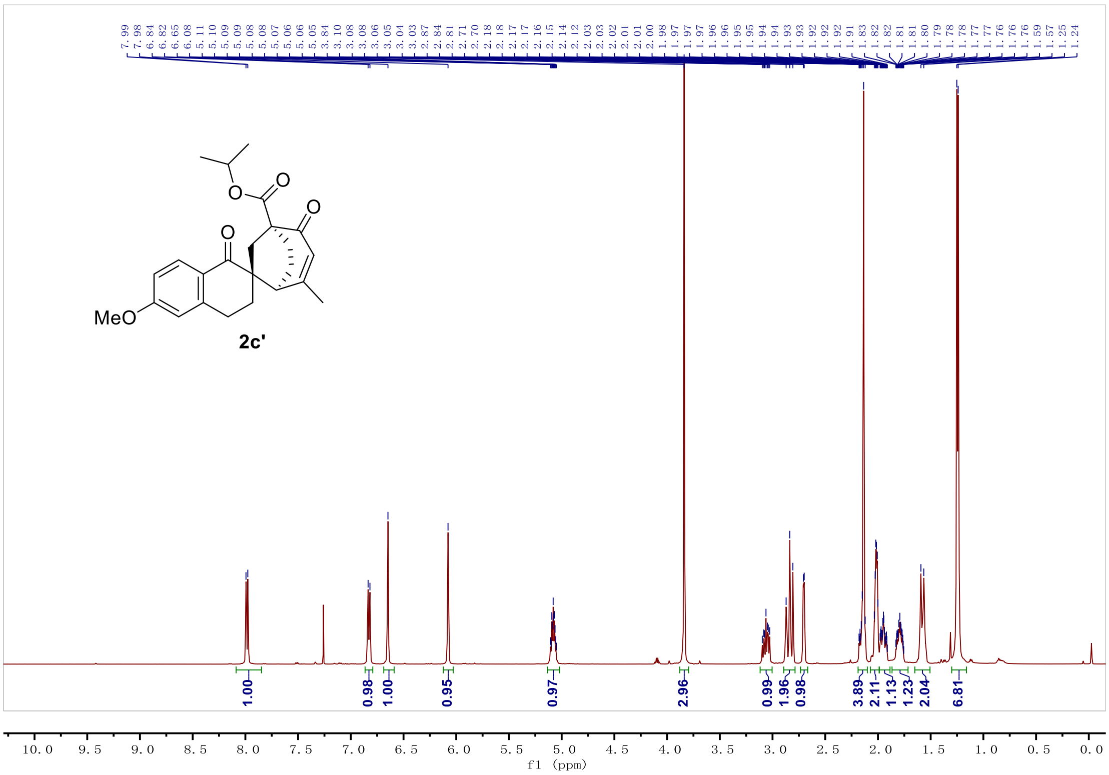

^{1}H NMR (500 MHz, CDCl3) spectrum of **2c′**

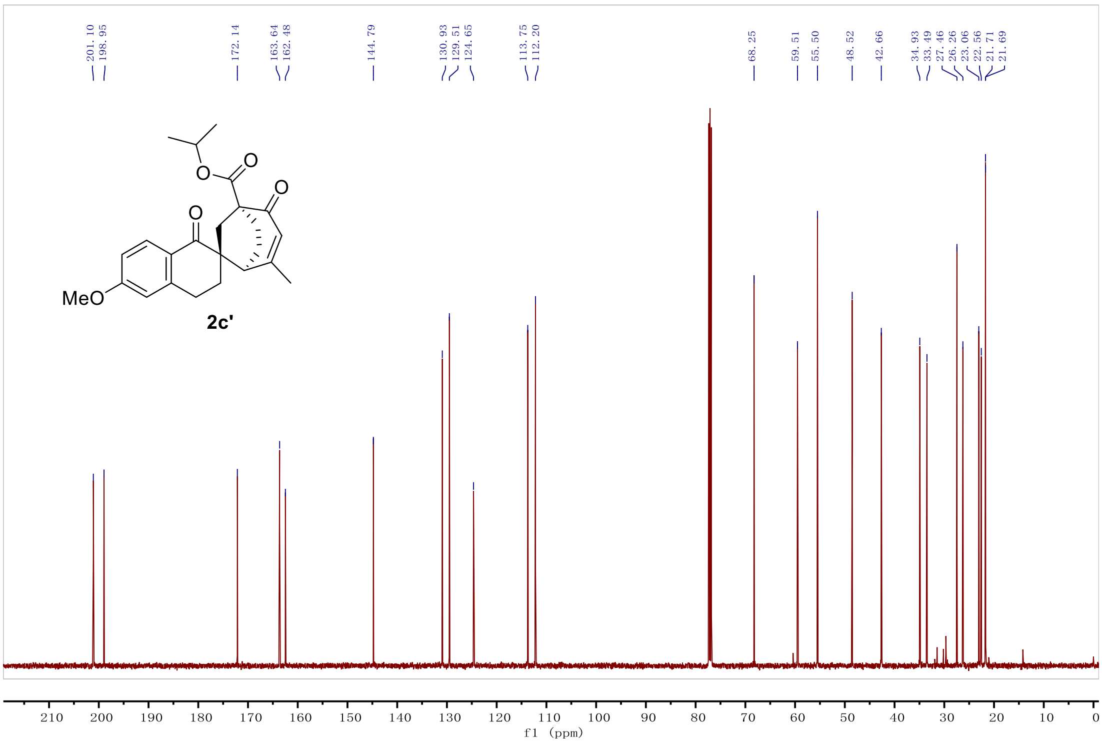

^{13}C NMR (126 MHz, CDCl3) spectrum of **2c′**

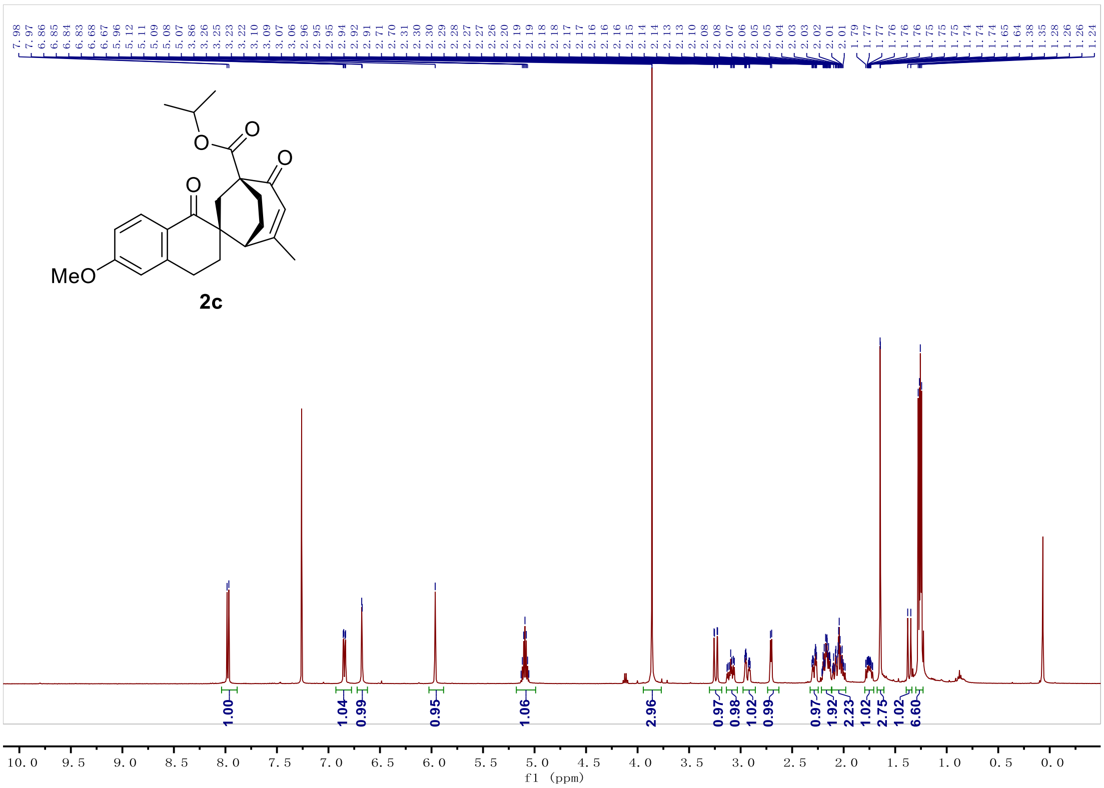

^{1}H NMR (500 MHz, CDCl3) spectrum o f **2c′**

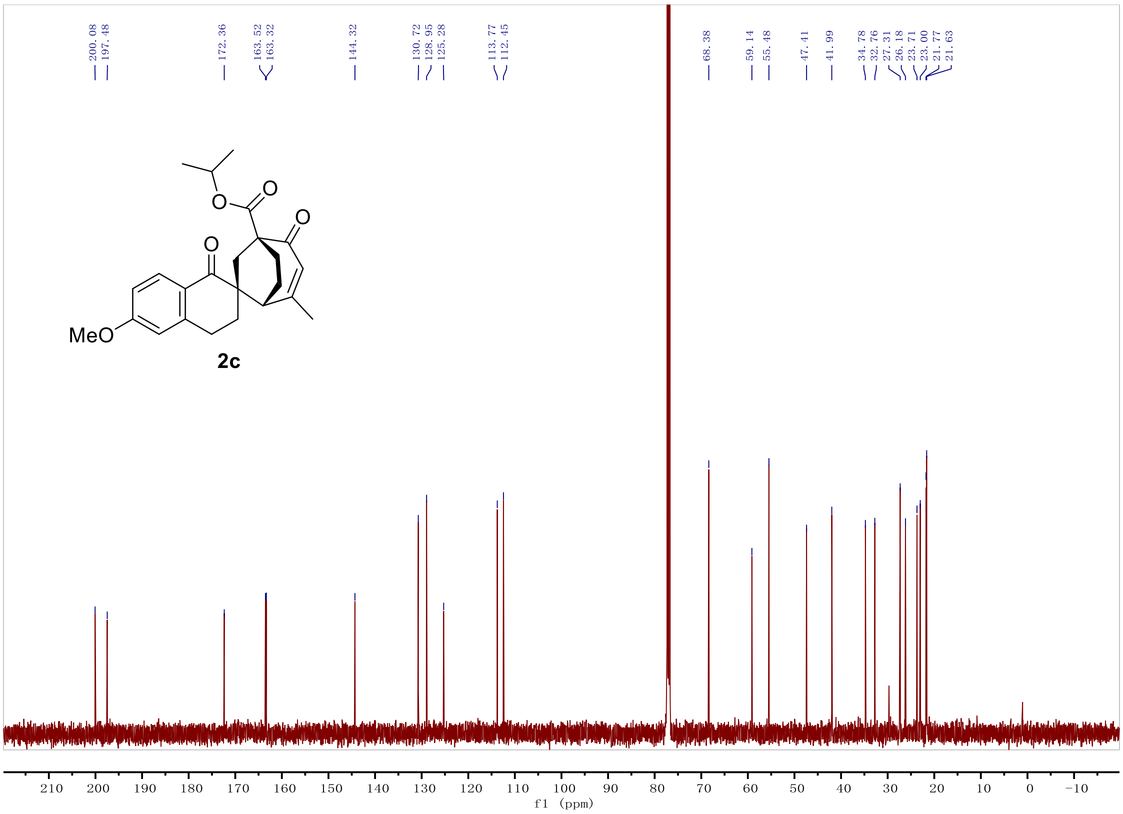

^{13}C NMR (126 MHz, CDCl3) spectrum of **2c′**

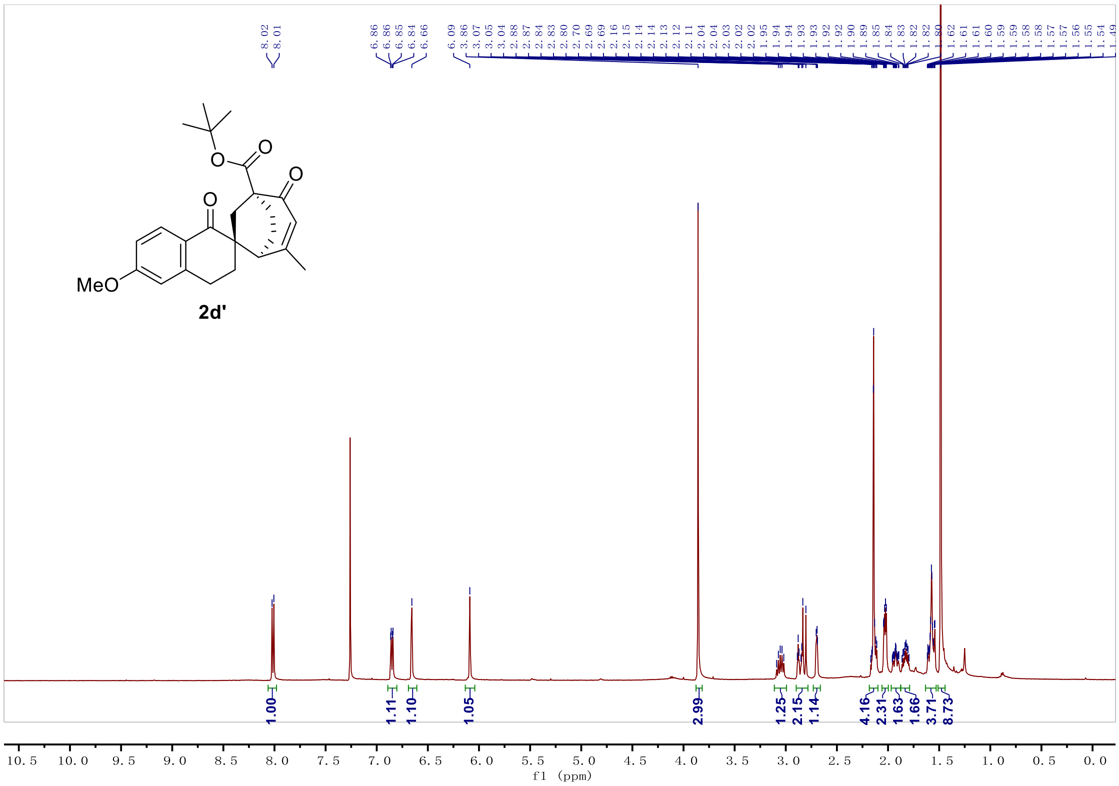

^{1}H NMR (500 MHz, CDCl3) spectrum of **2d′**

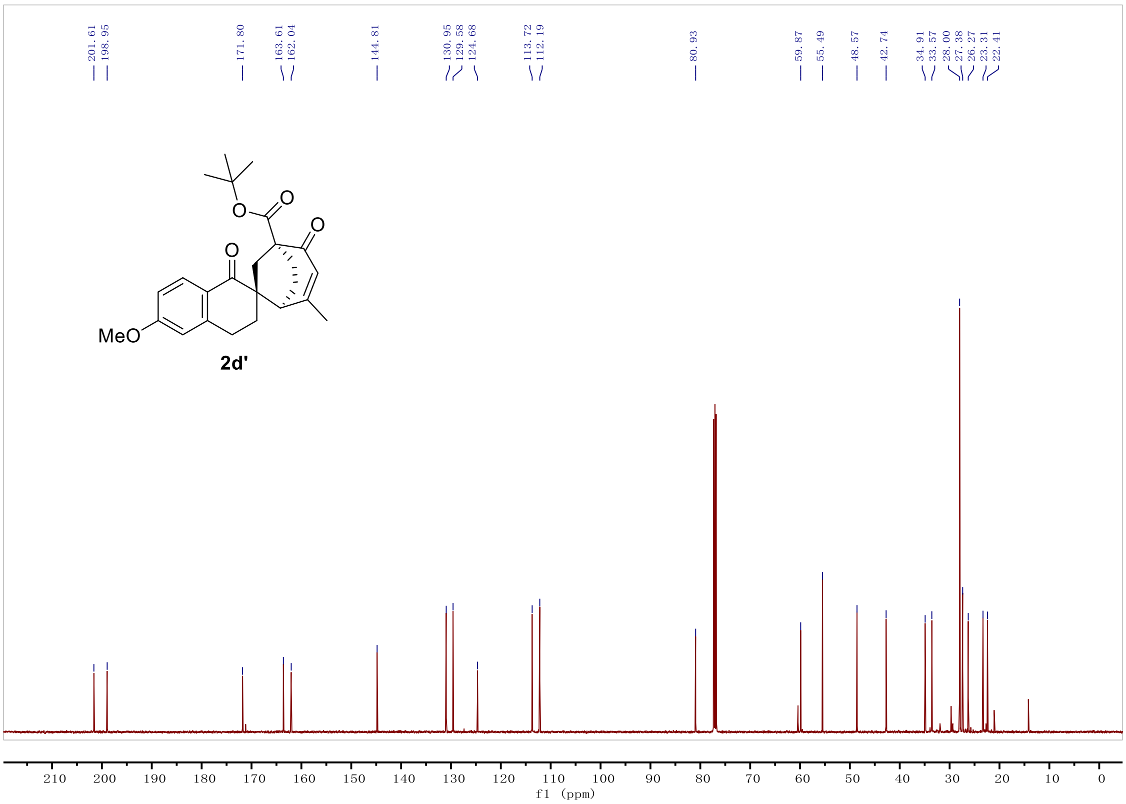

^{13}C NMR (126 MHz, CDCl3) spectrum of **2d′**

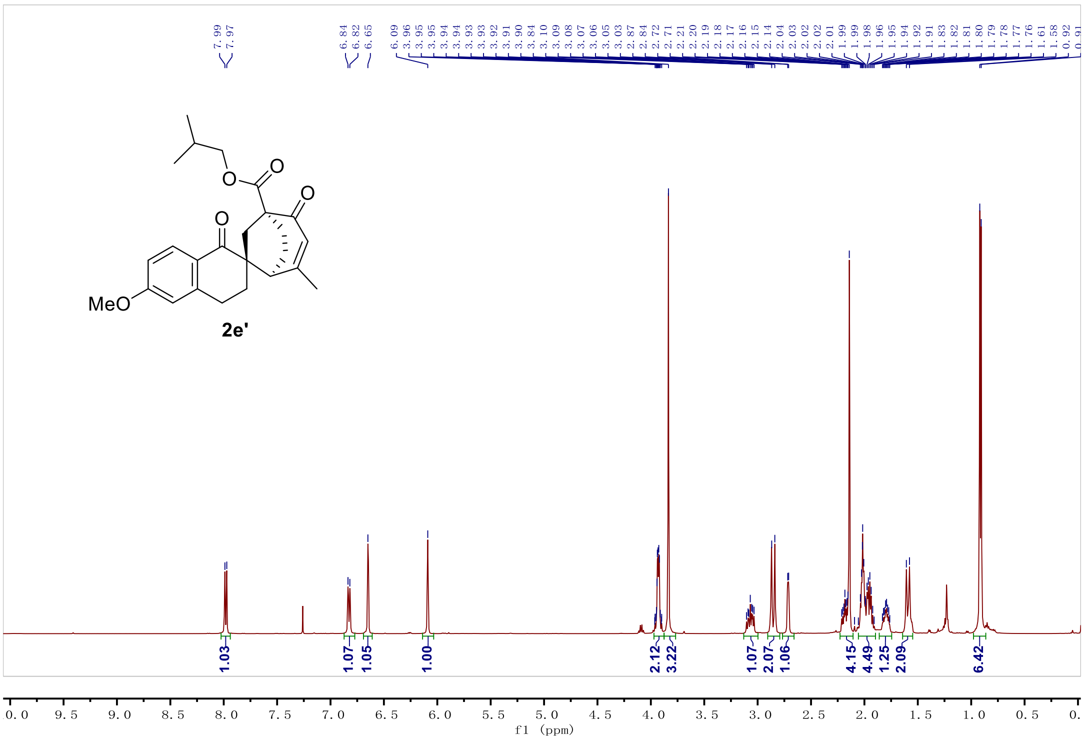

^{1}H NMR (500 MHz, CDCl3) spectrum **2e′**

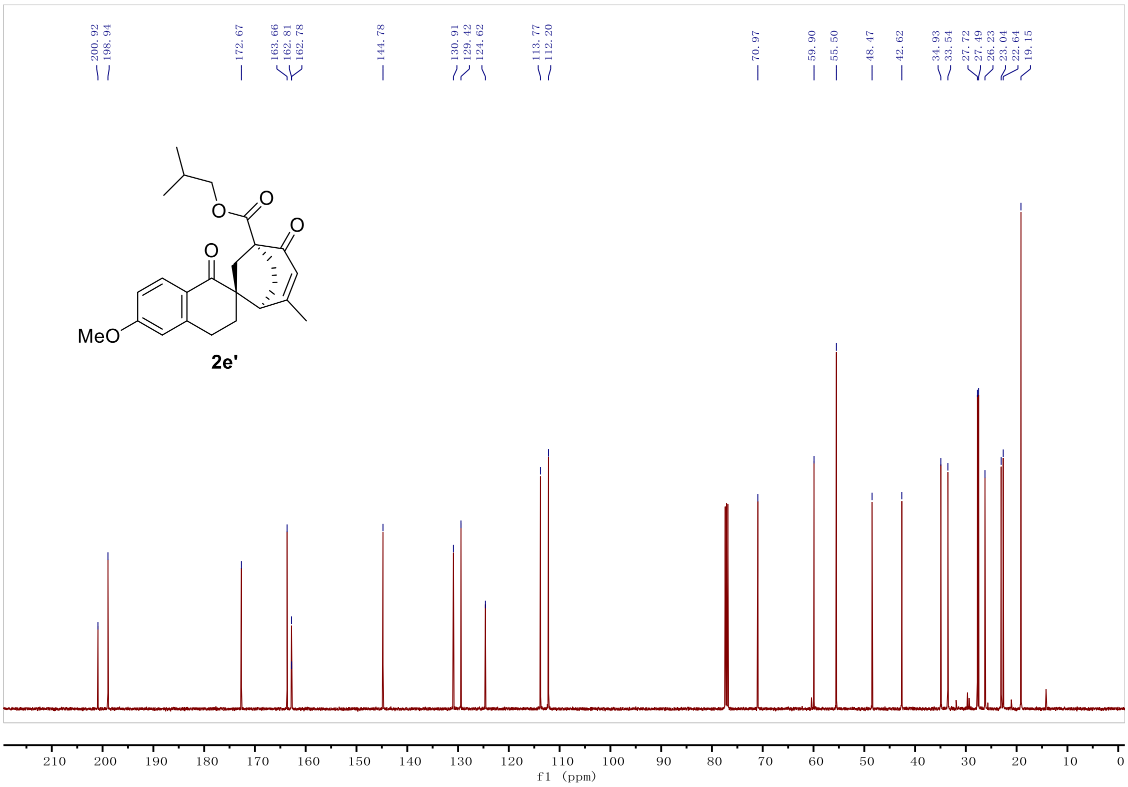

^{13}C NMR (126 MHz, CDCl3) spectrum of **2e′**
